# The role of RNF138 in DNA end resection is regulated by ubiquitylation and CDK phosphorylation

**DOI:** 10.1016/j.jbc.2024.105709

**Published:** 2024-02-01

**Authors:** Andrew J. Locke, Rabih Abou Farraj, Caroline Tran, Elham Zeinali, Fatemeh Mashayekhi, Jana Yasser Hafez Ali, J. N. Mark Glover, Ismail Hassan Ismail

**Affiliations:** 1 Division of Experimental Oncology, Department of Oncology, Faculty of Medicine & Dentistry, Cross Cancer Institute, University of Alberta, Edmonton, Alberta, Canada; 2 Department of Biochemistry, Faculty of Medicine & Dentistry, University of Alberta, Edmonton, Alberta, Canada; 3 Biophysics Department, Faculty of Science, Cairo University, Giza, Egypt

**Keywords:** RNF138, genomic instability, DNA repair, DNA end resection, homologous recombination, post-translational modifications, ubiquitylation, phosphorylation, cyclin-dependent kinases

## Abstract

Double-strand breaks (DSBs) are DNA lesions that pose a significant threat to genomic stability. The repair of DSBs by the homologous recombination (HR) pathway is preceded by DNA end resection, the 5′ to 3′ nucleolytic degradation of DNA away from the DSB. We and others previously identified a role for RNF138, a really interesting new gene finger E3 ubiquitin ligase, in stimulating DNA end resection and HR. Yet, little is known about how RNF138's function is regulated in the context of DSB repair. Here, we show that RNF138 is phosphorylated at residue T27 by cyclin-dependent kinase (CDK) activity during the S and G2 phases of the cell cycle. We also observe that RNF138 is ubiquitylated constitutively, with ubiquitylation occurring in part on residue K158 and rising during the S/G2 phases. Interestingly, RNF138 ubiquitylation decreases upon genotoxic stress. By mutating RNF138 at residues T27, K158, and the previously identified S124 ataxia telangiectasia mutated phosphorylation site (Han *et al.*, 2016, ref. 22), we find that post-translational modifications at all three positions mediate DSB repair. Cells expressing the T27A, K158R, and S124A variants of RNF138 are impaired in DNA end resection, HR activity, and are more sensitive to ionizing radiation compared to those expressing wildtype RNF138. Our findings shed more light on how RNF138 activity is controlled by the cell during HR.

DNA double-strand breaks (DSBs) occur when both DNA strands are severed in close proximity and are considered the most dangerous DNA lesion (1). They are mainly repaired by two major mechanisms, namely homologous recombination (HR) and non-homologous end joining (NHEJ). HR typically uses the sister chromatid as a template for repair (2). As the sister chromatid is generated upon DNA replication, this restricts HR activity to the S and G2 phases of the cell cycle (3, 4). HR commences with DNA end resection, the 5′ to 3′ nucleolytic degradation of DNA away from the DSB (5). The process generates 3′ single-stranded DNA (ssDNA) overhangs which are rapidly coated by replication protein A (RPA) complexes (5). End resection is initiated by the nuclease activities of the MRN (Mre11-Rad50-Nbs1) complex (5), the Mre11 endonuclease activity being activated upon binding to CtIP (6, 7, 8). The overhangs are further extended by the exonucleases Exo1 and Dna2 (5), after which RPA is exchanged for the Rad51 recombinase. Rad51 activity then drives the search for the homologous locus in the sister chromatid, initiating strand invasion and the eventual restoration of the site of damage by DNA synthesis (2).

If DSBs are instead repaired by NHEJ, the two DNA ends are directly ligated together once they are made to be chemically compatible by end processing (9). NHEJ requires the binding of the DNA ends by the Ku70-Ku80 (Ku) heterodimer (10), which serves as a platform to assemble the NHEJ machinery (9, 11, 12). NHEJ is active during all phases of the cell cycle (4), but is especially important in the G1 phase as that is when HR is not active. The decision of whether to perform NHEJ or HR is a dynamic process governed by multiple decision points (13). End resection biases cells to performing HR, as the resulting ssDNA overhangs are not amenable to ligation by NHEJ, and Ku itself has a low affinity to ssDNA (10). However, Ku recruits to DSBs regardless of the cell cycle phase (14), and its presence on chromatin is a block to end resection (15, 16). Thus, the removal of Ku is required for both end resection and HR to proceed (15, 16).

A recurring theme in the regulation of DSB repair is post-translational modification (PTM), the reversible covalent conjugation of protein or chemical groups onto biomolecules. PTMs include phosphorylation, catalyzed by kinases such as the cyclin-dependent kinases (CDKs) (17), and by members of the PI-3-kinase-related kinase (PIKK) family, such as ataxia telangiectasia mutated (ATM), which is activated by DNA damage (18). Ubiquitylation also plays a major role in the DSB response (19). Here, the ubiquitin protein is conjugated to its target substrates through the sequential activity of three classes of enzymes, E1, E2, and E3. We and others have shown that the E3 ubiquitin ligase RNF138 promotes HR (14, 20, 21, 22). Originally found to inhibit Wnt-β-catenin signaling (23), RNF138 belongs to a family of E3s with a similar domain structure (24). This includes an N-terminal really interesting new gene (RING) finger domain, which interacts with the E2 ubiquitin-conjugating enzyme (20, 23, 24), three zinc finger (ZNF) domains, and a C-terminal ubiquitin interacting motif (UIM). The zinc finger domains mediate its recruitment to DNA damage (14, 20, 22) and bind DNA (14, 20), showing a preference for ssDNA overhangs (14). Mechanistically, RNF138 promotes HR by stimulating DNA end resection (14, 20). It promotes the ubiquitylation of Ku80, which evicts Ku from chromatin (14). It also mediates the ubiquitylation of CtIP, facilitating CtIP’s accumulation at DSB sites (20). These parallel actions—promoting the recruitment of CtIP, a stimulator of resection, and facilitating the dissociation of Ku, which blocks resection—help ensure end resection can proceed (25). Downstream of end resection, a third target of RNF138-dependent ubiquitylation was found to be Rad51D (21, 22), a Rad51 paralogue that may contribute to Rad51 filament assembly (26). Although it is not clear how Rad51D ubiquitylation contributes to HR (21), the recruitment of Rad51D to DNA damage is dependent on RNF138 (22).

While it is established that RNF138 participates in HR, how its activity is controlled in HR has not been extensively investigated. Intriguingly, in response to ionizing radiation (IR), RNF138-dependent ubiquitylation of Ku80 occurs in S/G2 phase, but not G1 phase (14). This hints RNF138 activity might be under cell cycle regulation to coincide with the onset of HR. We also wondered if RNF138 could be regulated by ubiquitin conjugation. In this study, we find that RNF138 is phosphorylated in S and G2 phases by CDK activity on residue T27, and is also ubiquitylated on residue K158. Both sites mediate RNF138 function in DSB repair, as cells expressing the T27A and K158R mutants exhibit defective DNA end resection, HR activity, and heightened sensitivity to ionizing radiation relative to those expressing wildtype RNF138. We also investigate whether the same processes are impacted by mutations at S124, a previously identified ATM phosphorylation site on RNF138 (22). Our findings uncover how RNF138 activity is governed by the cell, providing more insight into its contribution to DSB repair.

## Results

### RNF138 protein expression is maintained over the course of the cell cycle

**Figure 1:**
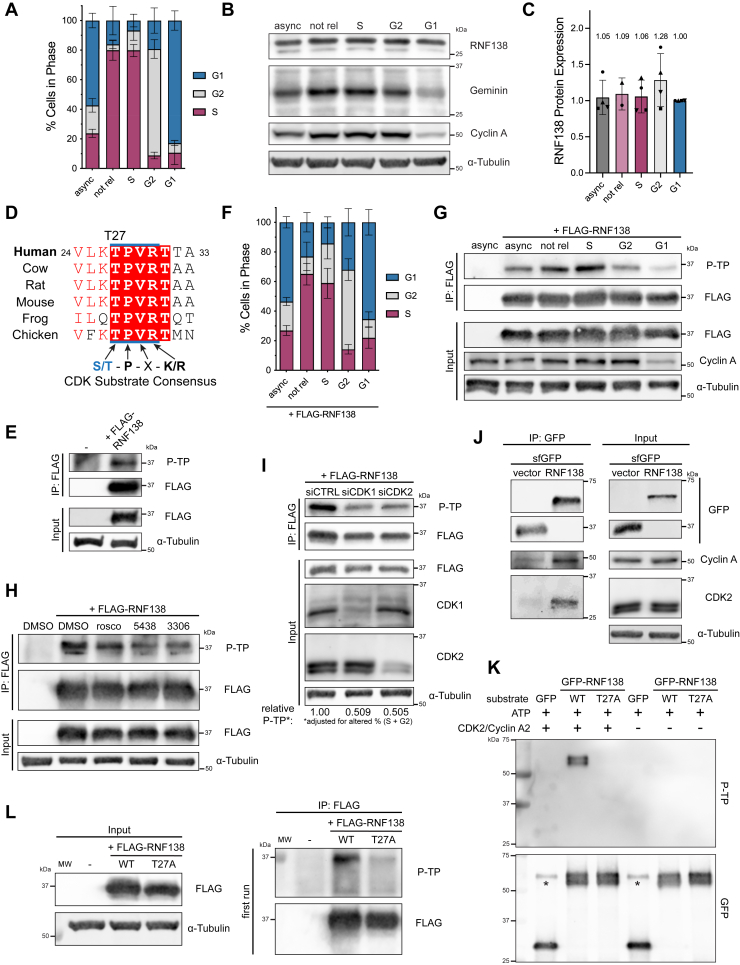
**RNF138 is Phosphorylated by CDK-Dependent Activity.***A*, flow cytometric analysis of propidium iodide signal in HeLa cells that were left asynchronous (async) or were synchronized by double-thymidine block without release (not rel) or with release to the S, G2, and G1 phases. *B*, immunoblot of whole cell extracts from cells treated as in (*A*). Geminin expression was used to confirm enrichment for cells in S phase, while Cyclin A expression was used to confirm enrichment in the S/G2 phases. *C*, quantification of endogenous RNF138 expression from (*B*). RNF138 expression was normalized to ⍺-tubulin levels. *D*, Clustal Omega amino acid sequence alignment of the CDK consensus phosphorylation motif from human RNF138 and its orthologues. UniProt accession numbers are indicated in Figure 4*A*. *E*, FLAG IP from asynchronous HeLa cells expressing FLAG-RNF138, immunoblotted for phosphorylated TP sites (P-TP). *F*, as in (*A*), but with HeLa transfected with FLAG-RNF138 during the first release step of double thymidine block. *G*, FLAG IP and IB of cells processed as in (*F*). *H*, FLAG-RNF138-expressing asynchronous HeLa cells treated with the CDK inhibitors roscovitine (rosco), AZD5438, or RO-3306 or vehicle control (DMSO) for 4 h before harvest, FLAG IP, and IB. *I*, HeLa cells were transfected with siRNA (si) to luciferase (CTRL), CDK1 or CDK2, along with FLAG-RNF138 DNA, and subjected to FLAG IP and IB. Quantification of the relative P-TP signal is adjusted for the altered proportion of cells in the S or G2 phase resulting from the knockdown of CDK1 or CDK2 from the same biological replicate (Fig. S3*C*). *J*, GFP co-IP for endogenous CDK2 and Cyclin A in HEK293 cells transfected with sfGFP-MAP-tagged (58) RNF138 or the empty vector (vector). *K*, CDK2 *in vitro* kinase assay, with GFP, GFP-RNF138-WT or -T27A as potential substrates. The GFP constructs were expressed in HEK293 cells and IP'd. Kinase activity was detected by immunoblotting for P-TP. ∗: a non-specific band detected by the GFP antibody. *L*, as in (*E*), but with FLAG-RNF138-WT and -T27A. The IP eluates were loaded at different volumes (first and second runs; the second run, with one-fourth the volume loaded, is shown in Fig. S3*D*). Shown are representative results from at least 2 (*I*), 3 (*G*, *H*, *K* and *L*), at least 3 (*E* and *J*), and 4 (*B*) biological replicates. Averages were calculated from at least 2 (*A*, *C*) or at least 5 (*F*) biological replicates pooled together. IP, immunoprecipitation; MW, molecular weight standards; sfGFP, superfolder green fluorescent protein; siRNA, short interfering RNA; WT, wildtype.

To ascertain how RNF138 is regulated, we first asked if its expression was controlled in a cell cycle-dependent manner. As the expression of the HR factor BRCA1 peaks during the S and G2 phases (27, 28, 29), we surmised RNF138 protein levels could behave similarly, coinciding with its role in mediating Ku80 ubiquitylation and eviction from chromatin in S/G2 (14). We chose to examine RNF138 expression in HeLa cells as they can be efficiently synchronized to the G1/S transition by double thymidine block. HeLa can then be released for different timepoints to approach specific cell cycle phases (30). Flow cytometric analysis confirmed that the chosen timepoints were sufficiently enriched for cells in the S, G2, or G1 phase (Fig. 1*A*). When whole cell extracts from these samples were immunoblotted, we detected a prominent immunoreactive band above 25 kDa in all cell cycle phases (Fig. 1*B*). As RNF138’s molecular weight is predicted to be 28 kDa, and transfecting cells with short interfering RNA (siRNA) targeting both coding and non-coding regions of the *RNF138* gene reduced detection of the band (Fig. S1*A*), the immunoblot signal just above 25 kDa represents endogenous RNF138. Interestingly, while a minor increase in RNF138 expression was seen at G2 phase, RNF138 was still adequately expressed in G1 phase, and overall, substantial changes in expression were not seen at any particular phase (Fig. 1*C*). We thus conclude that in HeLa cells, RNF138 protein expression is relatively constant over the course of the cell cycle.

### RNF138 is phosphorylated at residue T27 by CDK-dependent activity

We reasoned that RNF138 activity might be regulated during the cell cycle by a different mechanism. Transitions in the cell cycle are controlled by CDKs, whose activities accumulate at specific phases, and are stimulated when bound to their regulatory subunits, the cyclins (31, 32). CDK activity promotes HR and DNA end resection (33, 34, 35), and players in DNA end resection such as Mre11, Nbs1, CtIP, Dna2, and Exo1 are indeed targets of CDK phosphorylation (36, 37, 38, 39, 40, 41, 42, 43). Analysis of RNF138’s primary structure revealed a single putative CDK consensus phosphorylation motif (S/T-P-X-K/R) (32), with the potential phosphorylation site at threonine 27 (T27). This motif is conserved in RNF138 orthologues spanning *Xenopus laevis*, chicken, and mammals (Fig. 1*D*), and was predicted to be the only CDK site on RNF138 by the algorithm GPS 6.0 (44) (Fig. S1*B*). Further, the AlphaFold prediction of RNF138’s structure shows T27, despite being located within the RING domain, is solvent-accessible and potentially available for phosphorylation (Fig. S2, *A*–*E*). T27 is also at the centre of a positively charged surface of the RING (K26, R40, K41, R48, R80) (Fig. S2*B*), the addition of a phosphoryl group potentially altering electrostatics in the region. We thus hypothesized RNF138 is phosphorylated at residue T27 by CDK activity.

To uncover if RNF138 was phosphorylated, we first transfected HeLa cells with FLAG-tagged RNF138 and performed anti-FLAG immunoprecipitation to enrich for exogenous RNF138. Expressing FLAG-RNF138 yielded a protein that migrated at 37 kDa upon sodium dodecyl sulfate polyacrylamide gel electrophoresis (SDS-PAGE) (Fig. 1*E*), despite RNF138 being predicted to be 28 kDa. While endogenous RNF138 migrated at the expected position upon SDS-PAGE (Figs. 1*B* and S1*A*), immunoprecipitated FLAG-RNF138 was detected at 37 kDa by an anti-RNF138 antibody (Fig. S3*A*). We thus attribute the reduced electrophoretic mobility of FLAG-RNF138 to the FLAG tag, speculating the tag’s five negatively charged aspartate residues hinder SDS binding to the protein, impeding its migration during electrophoresis. We next immunoblotted the FLAG immunoprecipitates for the presence of phospho-threonine immediately N-terminal of a proline residue (phospho-Thr-Pro, or P-TP), a motif shared by the substrates of both CDKs and mitogen-activated protein kinases (MAPKs). We observed P-TP signal on FLAG-RNF138 (Fig. 1*E*), suggesting RNF138 was a substrate for phosphorylation at TP sites. To detect if RNF138 was phosphorylated in a cell cycle-dependent manner, we transfected HeLa cells with FLAG-RNF138 and synchronized them to S, G2, or G1 phase. The cells were suitably enriched for the phases of interest, although the synchronization efficiency was less than in untransfected HeLa cells (Fig. 1*F*, compare to Fig. 1*A*), likely from minor cytotoxicity resulting from FLAG-RNF138 overexpression. FLAG immunoprecipitation revealed P-TP signal that peaked at S phase and progressively weakened as cells approached the G2 and then G1 phases (Fig. 1*G*). In support of this, P-TP signal was partially reduced in cells that were not synchronized by double thymidine block (Fig. 1*G*), which primarily contain cells in G1 phase (Fig. 1*F*). Importantly, the P-TP signal occurred at 37 kDa, and did not appear in immunoprecipitates from untransfected cells (Fig. 1, *E* and *G*), indicating the P-TP signal was associated with FLAG-RNF138. We thus conclude RNF138 is phosphorylated in a cell cycle-dependent manner, with the modification occurring primarily in S and G2 phase.

CDK2 and CDK1 activity trigger progression through the S and G2 phases (31). To confirm that the P-TP signal observed on RNF138 was dependent on CDK activity, we treated HeLa cells expressing FLAG-RNF138 with roscovitine and AZD5438, inhibitors that target both CDK1 and CDK2 activity (45, 46), and RO-3306, an inhibitor of CDK1 activity (47). The P-TP signal in FLAG immunoprecipitates was reduced upon treatment with each inhibitor (Fig. 1*H*). As a control, treating the cells with SB203580, an inhibitor of MAPK activity, did not affect the P-TP signal, indicating the phosphorylation on RNF138 arose solely from CDK-dependent activity (Fig. S3*B*). We also assessed the P-TP signal on FLAG-RNF138 when cells were transfected with siRNA targeting CDK1 or CDK2. Knocking down either kinase capably reduced RNF138 TP phosphorylation (Fig. 1*I*). While CDK1 or CDK2 depletion did decrease the proportion of cells in S or G2 phase, in both cases from 49.5% to ∼45% (Fig. S3*C*), accounting for this difference still resulted in the P-TP signal dropping ∼50% when either CDK was depleted (Fig. 1*I*). Thus, the P-TP signal on RNF138 is dependent on CDK1 and CDK2. In support of a role for CDK2 in RNF138 phosphorylation, CDK2 and its binding partner Cyclin A could co-immunoprecipitate with RNF138 (Fig. 1*J*), indicating RNF138 may form a complex with CDK2-Cyclin A. Consistent with this, GFP-RNF138 was capably phosphorylated by CDK2/Cyclin A2 *in vitro*. Moreover, compared to GFP-RNF138, GFP itself was only minimally phosphorylated by CDK2/Cyclin A2, demonstrating the specificity of CDK2 activity toward RNF138 (Fig. 1*K*). Overall, our findings suggest RNF138 is phosphorylated by the action of CDK1 and CDK2.

To demonstrate that T27 is the site of TP phosphorylation on RNF138, we ablated the site by mutating T27 to a non-phosphorylatable alanine residue (T27A) in the FLAG-RNF138 construct. P-TP signal was severely impaired in immunoprecipitates of T27A relative to wildtype (WT) FLAG-RNF138 (Fig. 1*L*). At first, we could not completely eliminate P-TP signal from the T27A mutant (Fig. 1*L*, right panel), speculating residual signal arises from other proteins co-precipitating with FLAG-RNF138 at the same molecular weight. In line with this, loading a smaller amount of the immunoprecipitates for SDS-PAGE completely abrogated P-TP signal in the T27A mutant (Fig. S3*D*). As well, unlike GFP-RNF138-WT, the T27A variant was not phosphorylatable by CDK2/Cyclin A2 *in vitro* (Fig. 1*K*). Altogether, our results suggest RNF138 is phosphorylated at position T27 in a CDK1- and CDK2-dependent manner.

### RNF138 is a target for polyubiquitylation

The repair of DSBs is coordinated by a cascade of ubiquitylation events, contributing to protein recruitment to sites of damage, the assembly and disassembly of complexes involved in repair, and protein turnover (19, 48). We consequently were curious if RNF138 was also a target of ubiquitylation. Previously, we studied SUMOylation of CtIP in HeLa cells stably expressing His-tagged SUMO-2; SUMO-2 is a ubiquitin-like modifier also conjugated to proteins (49). From these cells, we isolated the pool of His-tagged proteins by nickel affinity purification (“His pull-down”), which represented those that had incorporated SUMO-2. We attempted to use the same strategy to study the ubiquitylation of endogenous RNF138, this time using HeLa cells stably expressing 6xHis-biotin-tagged ubiquitin (HeLa HB-ubiquitin) (50), with the goal of exploiting the 6xHis component for nickel affinity purification. Nickel beads could successfully enrich ubiquitylated proteins from extracts of these cells relative to plain HeLa cells not expressing HB-ubiquitin (Fig. S3*E*). However, despite our best efforts, we were unable to detect ubiquitylated RNF138 in the His pull-down fraction, even though RNF138 expression was observed in whole cell extracts (Fig. S3*E*, rightmost panel). Monoubiquitylated RNF138 should increase in molecular weight by ∼10 kDa, and such a species (∼38 kDa) was not clearly observed in HeLa HB-ubiquitin relative to plain HeLa cells (Fig. S3*E*). Perhaps ubiquitylated RNF138 is of low abundance, such that these species are below the detection limit of our immunoblotting approach. The issue is exacerbated by RNF138 antibodies detecting non-specific signals during immunoblotting (Fig. S3*E*, rightmost panel). It is difficult to discern which higher order species in the His pull-down arise from ubiquitylated RNF138 or are simply non-specific in nature, complicating our analysis. Thus, in our hands, we do not consider His pull-down a viable approach to study RNF138 ubiquitylation.

**Figure 2:**
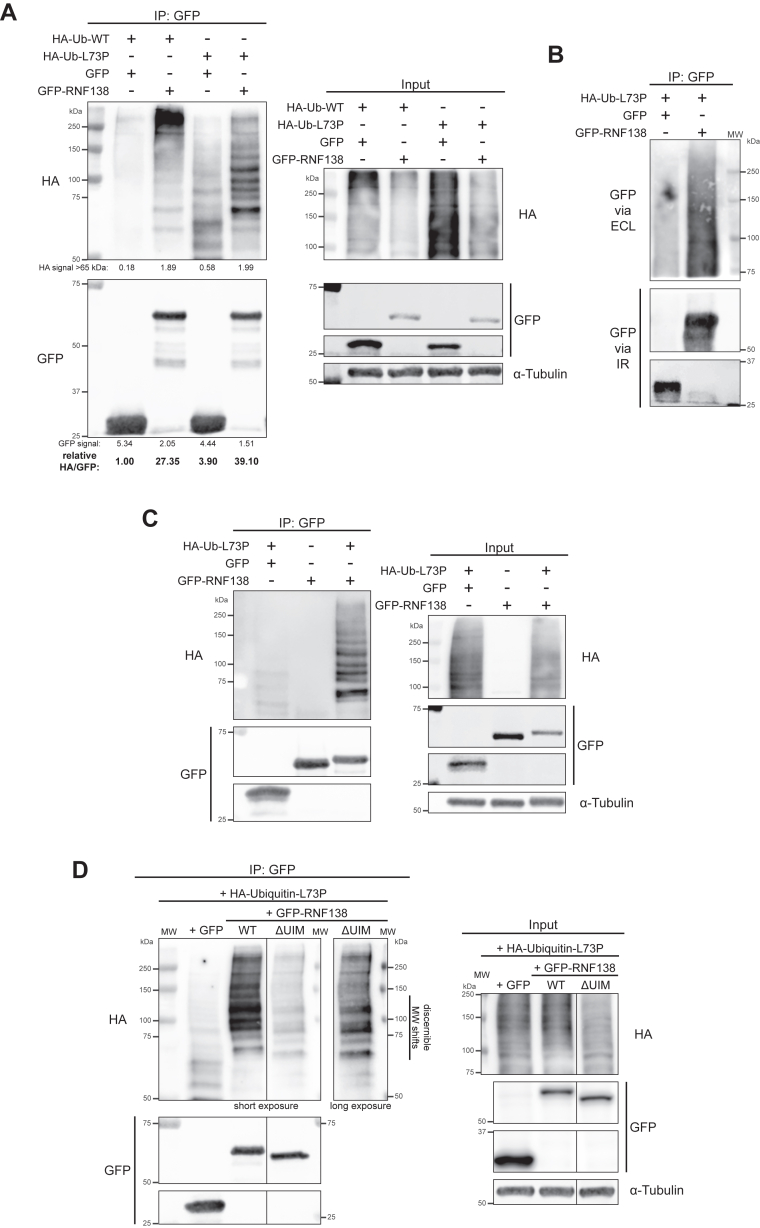
**RNF138 is Constitutively Polyubiquitylated.***A*–*D,* stringent GFP IP were performed for HEK293 cells expressing (*A* and *B*) HA-ubiquitin-WT or -L73P and GFP-RNF138 or free GFP, (*C*) GFP or GFP-RNF138 with or without HA-Ub-L73P, or (*D*) HA-Ub-L73P with GFP or GFP-RNF138-WT or -ΔUIM, the products of which were then immunoblotted. *B*, contains select IP samples from (*A*), with GFP immunoblot signals below 75 kDa developed by infrared-fluorescent (IR) secondary antibodies, and GFP signals above 75 kDa developed by horseradish peroxidase-coupled secondary antibodies and enhanced chemiluminescence (ECL). Shown are representative results from at least four biological replicates (each of *A*, *C*, *D*). IP, immunoprecipitation; MW, molecular weight standards; WT, wildtype.

As an alternate strategy to detect RNF138 ubiquitylation, we turned to exogenous co-expression of green fluorescent protein (GFP)-tagged RNF138 and HA-tagged ubiquitin (HA-Ub) in cells followed by immunoprecipitation for GFP. This approach would boost the expression of both ubiquitin and RNF138, improving the detection of ubiquitylated RNF138. In addition to WT-ubiquitin, we also used an HA-tagged mutant, with leucine 73 substituted with proline (HA-Ub-L73P), that is conjugable to substrates but resistant to deubiquitinating enzymes (51). We hoped this would stabilize ubiquitylated RNF138, increasing the likelihood of its detection. We also performed these experiments in HEK293 cells, which capably tolerated overexpression of GFP-RNF138. GFP-RNF138 was isolated by immunoprecipitation with anti-GFP beads under stringent conditions. Eluates from the beads were then processed for SDS-PAGE and subjected to immunoblotting for the HA tag. When HA-Ub-WT and GFP-RNF138 were co-expressed, we detected a smear of immunoreactivity to HA appearing under 75 kDa and extending beyond 250 kDa, where the signal greatly intensified (Fig. 2*A*), strongly suggesting GFP-RNF138 (theoretically ∼55 kDa in size but migrating at ∼63 kDa to start) was polyubiquitylated. This signal was absent in cells when HA-Ub-WT was co-expressed with empty vector GFP (Fig. 2*A*). Notably, higher order HA signal was also present when the same experiment was performed with HA-Ub-L73P (Fig. 2*A*). Similarly, this signal was much reduced when GFP was used in place of GFP-RNF138. However, using HA-Ub-L73P shifted the bulk of the HA smear between <75 to ∼150 kDa, and reduced the intensity of the signal appearing >250 kDa (Fig. 2*A*). At lower molecular weights, the smear resolved to a distinct laddering pattern perhaps revealing different species of ubiquitylated RNF138, each band suggestive of a different number of ubiquitin moieties attached to the protein. The discrepancy in how RNF138 conjugates appear using HA-Ub-WT or -L73P is consistent with the observation that L73P-Ub is conjugated to targets less efficiently than WT-Ub *in vitro* (51). On ubiquitin, L73 sits in a hydrophobic patch used by some E3 ligases to assemble polyubiquitin chains, and the proline substitution disrupts hydrophobic packing in this region (51). In our case, it is clear expressing L73P- instead of WT-Ub restricts the extent by which RNF138 can be polyubiquitylated, but it is still polyubiquitylated nonetheless. While smearing above unmodified GFP-RNF138 did not appear when we first re-probed the immunoprecipitates for GFP (Fig. 2*A*), these immunoblots were performed using infrared-fluorescent secondary antibodies, which we find are less sensitive to signals of low abundance. When chemiluminescence was instead used to develop the GFP immunoblots, we observed smearing above the unconjugated GFP-RNF138 band, although the laddering was not as distinct as that detected by HA immunoblotting (Fig. 2*B*).

Continuing on, to confirm RNF138 is ubiquitylated, we observed that the ladder of HA signal in immunoprecipitates of GFP-RNF138 was absent if HA-Ub-L73P was not transfected into cells (Fig. 2*C*), indicating the signal resulted from HA-Ub-L73P co-expression. We also co-expressed a truncated GFP-RNF138 deleted of its UIM (ΔUIM) (Fig. S3*F*) with HA-Ub-L73P. Here, the bands in the HA-Ub ladder exhibited a downward shift in size relative to those from GFP-RNF138-WT, consistent with the decrease in molecular weight resulting from deleting the UIM (Fig. 2*D*). This definitively showed that the HA signal on GFP-RNF138 arises from ubiquitylation events on RNF138 itself, and not from proteins that are inadvertently co-immunoprecipitated. We thus conclude that RNF138 is constitutively polyubiquitylated in cells.

### Insights into the dynamics of RNF138 ubiquitylation

**Figure 3:**
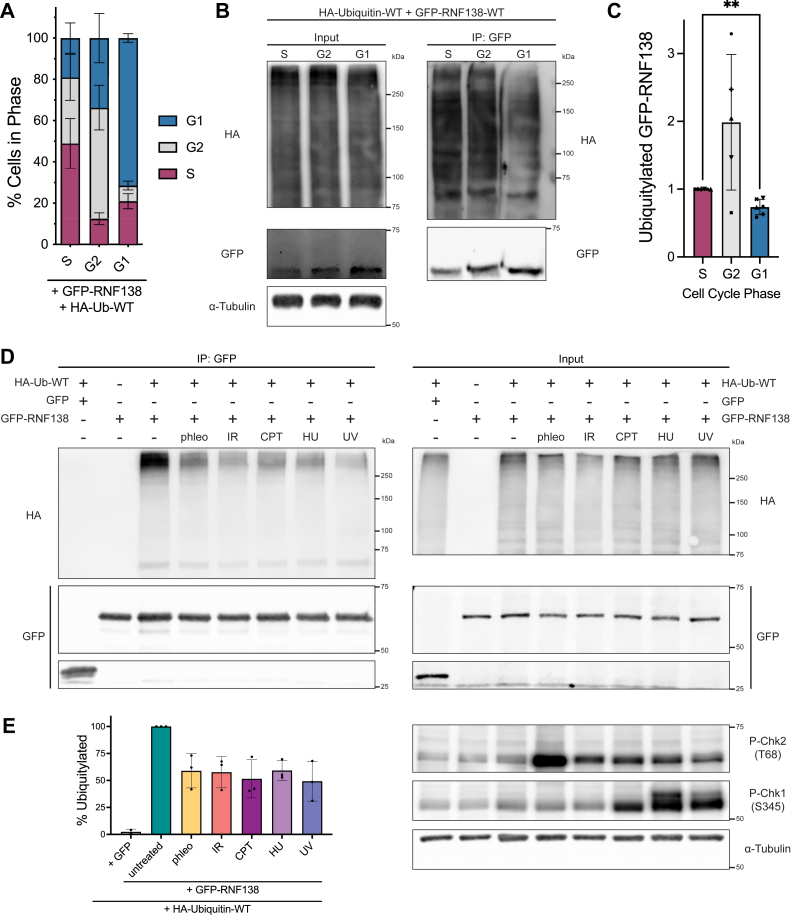
**The Dynamics of RNF138 Ubiquitylation Upon Cell Cycle Progression and DNA Damage.***A*, flow cytometric analysis of propidium iodide signal in HeLa cells synchronized by double-thymidine block and release. The cells were transfected with GFP-RNF138 and HA-ubiquitin-WT during the first release. *B*, GFP IP assay from cells treated as in (*A*). *C*, quantification of ubiquitylated GFP-RNF138 from (*B*). HA signal in the IP fraction was normalized to the GFP signal in the IP fraction. ∗∗: a paired, two-tailed, parametric *t* test comparing the S and G1 phase conditions for ubiquitylated GFP-RNF138 gives *p* = 0.0043 (*p* ≤ 0.01). *D*, HEK293 cells were transfected with HA-ubiquitin-WT and GFP or GFP-RNF138 and exposed to phleomycin (phleo), IR, CPT, hydroxyurea (HU), or ultraviolet light (UV). The cells were then subjected to GFP IP and immunoblot analysis. Chk1 and Chk2 phosphorylation (P) at S345 and T68, respectively, are markers for activation of the DNA damage response. *E*, quantification of (*D*) as per (*C*). Shown are representative results from 3 (*D*) and at least 6 (*B*) biological replicates. Averages were calculated from 3 (*E*), at least 4 (*A*), or at least 5 (*C*) biological replicates pooled together. CPT, camptothecin; IP, immunoprecipitation; IR, ionizing radiation; WT, wildtype.

Having determined that RNF138 is a target for ubiquitylation, we sought to understand how different cellular conditions would affect RNF138 ubiquitylation. We first asked if the cell cycle factored into the degree of RNF138 ubiquitylation. We synchronized HeLa cells to the S, G2, and G1 phases, and during the process co-expressed in them low levels of HA-Ub-WT and GFP-RNF138. Despite transfecting in low levels of GFP-RNF138, its expression was slightly cytotoxic to HeLa, resulting in a reduction in synchronization efficiency (Fig. 3*A*, compare with Fig. 1*A*). Still, GFP immunoprecipitation consistently revealed more RNF138 ubiquitylation in samples enriched for S phase cells compared to ones enriched for G1 phase cells (Fig. 3, *B* and *C*). As well, samples enriched for cells in G2 had similar or greater levels of ubiquitylation than those enriched for S phase cells (on average, 1.986 times more). We infer then that RNF138 ubiquitylation is cell cycle-dependent, with ubiquitylation increasing in S and G2 phase and decreasing in G1 phase.

We also examined if genotoxic stress would alter RNF138 ubiquitylation. We expressed HA-Ub-WT and GFP-RNF138 in HEK293 cells and treated the cells with various DNA-damaging agents. We used ionizing radiation from a gamma source (IR) and phleomycin (phleo) to induce DSBs. We also treated cells with ultraviolet light (UV), along with the replication stress-inducing agents camptothecin (CPT, which also induces DSBs) and hydroxyurea (HU). Treating cells with any of the agents reduced the signal of higher-order HA-Ub on GFP-RNF138 (Fig. 3, *D* and *E*). This was not due to variable expression of HA-Ub-WT between the samples, as signals from HA-Ub-WT conjugates were comparable in whole cell extracts (Fig. 3*D*). Therefore, DNA damage induces a reduction in RNF138 ubiquitylation. Overall, our data reveal differential ubiquitylation of RNF138 depending on cell status, with ubiquitylation rising when cells are in S/G2 phase and lessening upon genotoxic stress.

### K158 is a site of RNF138 ubiquitylation

**Figure 4:**
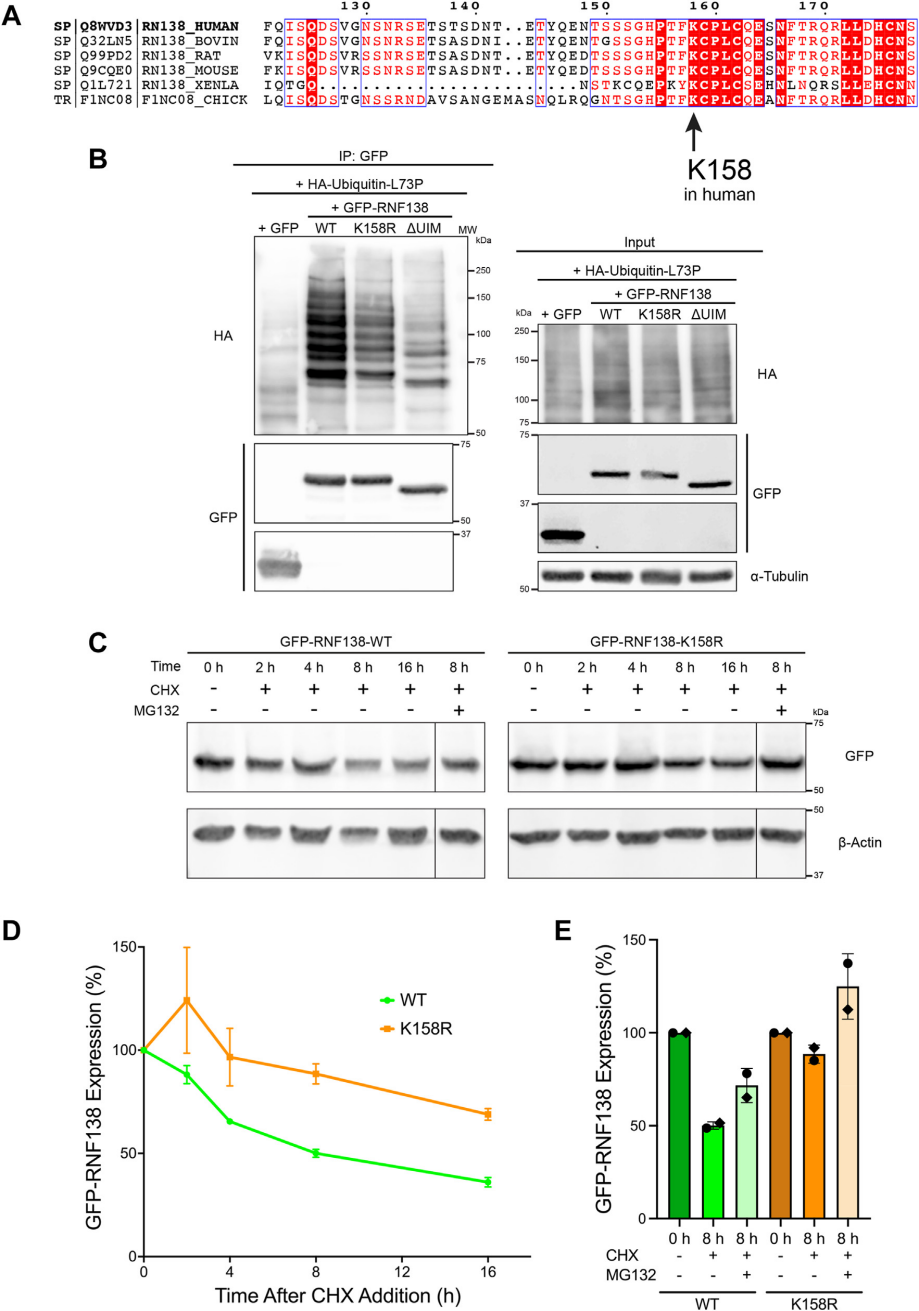
**K158 is a Site of RNF138 Ubiquitylation.***A*, Clustal Omega amino acid sequence alignment of the region containing the K158 residue from human RNF138 with its orthologues. *B*, stringent GFP IP was performed for HEK293 cells transfected with HA-Ub-L73P and GFP or GFP-RNF138-WT, -K158R, or -ΔUIM and then immunoblotted. *C*, immunoblot of whole cell extracts of HEK293 cells transfected with GFP-RNF138-WT or -K158R and treated with cycloheximide (CHX) with or without MG132 for various timepoints. *D* and *E*, quantifications of (*C*). Shown are representative results from 2 (*C*) and at least 5 (*B*) biological replicates. Averages in (*D*) and (*E*) are of two biological replicates pooled together. IP, immunoprecipitation; MW, molecular weight standards; WT, wildtype.

Four proteomic screens have identified residue K158 as a ubiquitylation site on RNF138 expressed in HEK293, HeLa cells, and Jurkat cells (52, 53, 54, 55). K158 was also a site of ubiquitylation in a study that found RNF138 promotes oncogenic signaling in lymphomas expressing L265P-mutated MyD88 (56). Interestingly, the K158 residue is conserved across RNF138 orthologues (Fig. 4*A*). It is also predicted to be solvent-exposed (Fig. S2, *A*, *C*–*E*), in line with it being accessible for modification. To determine if K158 was indeed a ubiquitylation site in our system, we generated a K158R mutant of GFP-RNF138, the arginine substitution maintaining the positive charge but ablating the site of ubiquitylation. GFP immunoprecipitates of the construct exhibited a notable reduction in HA-Ub signal relative to WT-RNF138 when co-expressed with HA-Ub-L73P (Fig. 4*B*), substantiating K158 as a site for RNF138 ubiquitylation.

As polyubiquitin chains are known to target proteins for degradation by the proteasome (57), we determined the impact of disrupting K158 ubiquitylation on RNF138 stability by a cycloheximide chase assay. We expressed GFP-RNF138-WT or -K158R in HEK293 cells, inhibited protein synthesis with cycloheximide, and monitored exogenous RNF138 levels over time. We found that the K158R mutant was turned over at a reduced rate compared to WT-RNF138 (Fig. 4, *C* and *D*). In the same experiment, we also inhibited proteasome activity with the compound MG132. MG132 treatment partially and fully restored WT- and K158R-RNF138 stability, respectively (Fig. 4, *C* and *E*), indicating RNF138 turnover is at least partly dependent on proteasomal activity. Taken together, these findings indicate K158 is a site of constitutive ubiquitylation on RNF138, and modification of this site by ubiquitin can promote proteasome-mediated turnover of the protein.

### T27 and K158 are not required for the recruitment of RNF138 to sites of damage

**Figure 5:**
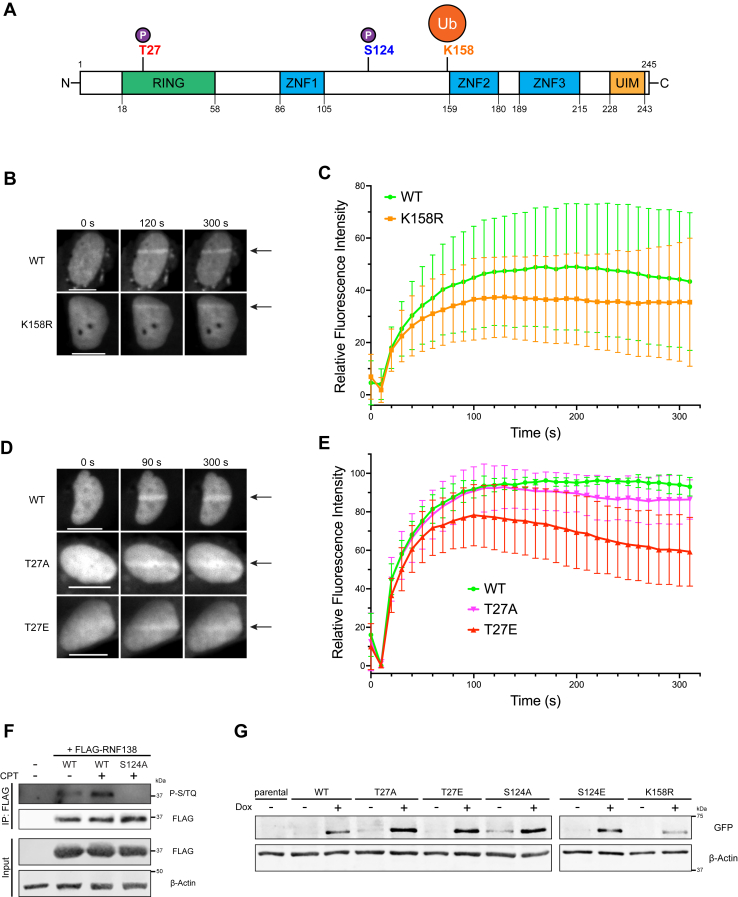
**Recruitment Kinetics of RNF138 Mutants at T27 and K158 to Sites ofDNADamage; Stable Expression of T27, S124, and K158 Variants in U2OS-TREx Cells.***A*, schematic diagram of the structural domains in wildtype (WT) RNF138 and the positions of the post-translational modification sites investigated. *B*, representative micrographs of live U2OS cells transfected with GFP-RNF138-WT or -K158, microirradiated in a line across the nucleus with a 405 nm laser, and monitored by time-lapse microscopy (arrow: region irradiated). *C*, quantification of relative fluorescence intensity over time at the microirradiated region in (*B*). The relative recruitment kinetics of 30 cells per construct, pooled from three biological replicates, were averaged. *D*, as in (*B*) but for U2OS expressing GFP-RNF138-WT, -T27A, and -T27E. *E*, quantification of relative fluorescence intensity over time at the microirradiated region in (*D*). For a single replicate, the mean recruitment kinetics of ∼25 cells were calculated for each construct. The normalized means from three biological replicates were then averaged and plotted (∼75 cells total per construct). *F*, FLAG IP and subsequent immunoblot of HeLa cells expressing FLAG-RNF138-WT or -S124A and treated with CPT or not for 1 h. *G*, IB of parental or stable sfGFP-RNF138 variant-expressing U2OS-TREx cells induced with doxycycline (Dox) or not. Shown are representative results from 3 (*B* and *D*), 4 (*F*), and at least 7 (*G*) biological replicates. Scale bars denote 10 μm. CPT, camptothecin; IP, immunoprecipitation; sfGFP, superfolder green fluorescent protein; TREx, tetracycline-regulated expression; WT, wildtype.

We so far observed CDK-dependent phosphorylation of RNF138 on residue T27 and, in support of previous findings (52, 53, 54, 55, 56), ubiquitylation on RNF138 at K158. To study how these post-translational modifications impact RNF138’s function in the DSB response, we continued our investigations in U2OS osteosarcoma cells, a standard cell line used to study the DSB response. The three zinc finger domains (ZNF1, ZNF2, and ZNF3) are together essential for targeting RNF138 to DNA damage sites (14). ZNF2 and ZNF3 are predicted to pack together in a single domain; this ZNF2/3 domain is flexibly tethered to the rest of the protein by a long linker (Fig. S2, *A*, *C*–*E*). As K158 is situated immediately N-terminal of ZNF2 in RNF138 (Fig. 5*A*), we asked if ubiquitylation at K158 might contribute to RNF138 recruitment to DNA damage. We transiently transfected U2OS cells with minimal amounts of DNA encoding GFP-RNF138-WT or -K158R. To induce DNA damage, we pre-sensitized cells with Hoescht 33358, then irradiated nuclei with a stripe of 405 nm light. Timelapse fluorescence imaging was used to monitor the recruitment of the fusion proteins to the regions of damage over 5 min. Both GFP-RNF138-WT and -K158R effectively accumulated at laser stripes (Fig. 5*B*), although the K158R mutant exhibited a minor impairment when the recruitment kinetics were examined (∼25% decrease in signal relative to WT at 200 s) (Fig. 5*C*). We conclude then that the K158 site promotes but is not essential for RNF138’s accumulation on damaged DNA.

We next asked if T27 played a role in RNF138 recruitment to damage. This time, we generated phospho-ablating alanine (T27A) and phospho-mimicking glutamate (T27→E27, T27E) substitutions for T27 in the context of GFP-RNF138. While the WT and T27A GFP-RNF138 constructs were capably enriched at laser-microirradiated stripes (Fig. 5*D*), the T27A mutant displayed a subtle defect in retention over time (∼10% decrease in signal relative to WT at 240 s) (Fig. 5*E*). Meanwhile, the T27E variant accumulated at laser stripes, but exhibited a slight defect in accrual (14.75% decrease in signal relative to WT at 100 s) and a discernible defect in retention (36.22% decrease in signal relative to WT at 300 s) (Fig. 5, *D* and *E*). It appears then that a permanent negative charge at T27 may impact sustained localization of RNF138 to chromatin, perhaps owing to electrostatic repulsion impacting DNA binding potentially contributed by the positively charged surface of the RING domain (Fig. S2, *A* and *B*). Regardless, it is clear the function of residue T27 is not required for RNF138’s localization to sites of DNA damage. Collectively, these data suggest modifications at K158 and T27 are not required for RNF138’s accrual at DNA damage, but may have minor roles in promoting recruitment to (K158) and regulating retention on (T27) chromatin.

### The RNF138 PTM sites T27, S124, and K158 are important for DNA end resection

An additional residue on RNF138, S124, has been reported to be phosphorylated in an ATM kinase- and DNA damage-dependent manner (22). S124 resides in the long, flexible linker between ZNF1 and ZNF2 (Figs. 5*A* and S2, *A*, *C*–*E*), so it would also be available for phosphorylation. We confirmed that S124 was the sole DNA damage-induced phosphorylation site on RNF138 by immunoblotting immunoprecipitates of FLAG-RNF138-WT and -S124A for the phosphorylated PIKK substrate consensus motif (phospho-Ser/Thr-Gln, or P-S/TQ) (Fig. 5*F*). Treating HeLa cells with CPT induced S/TQ phosphorylation on FLAG-RNF138-WT, while this signal was completely abolished in the S124A mutant. While it has been reported that the S124A mutant is proficient in recruitment to laser-microirradiated stripes (22), the effects of S124 phosphorylation on other aspects of HR have not been investigated.

As RNF138 promotes DNA end resection in HR (14, 20), we next examined what impact mutations at T27, S124, and K158 would have on the efficiency of DNA end resection. We opted for a simultaneous knockdown and complementation approach in U2OS cells, depleting endogenous RNF138 and re-expressing exogenous, siRNA-resistant WT-RNF138 or versions where the PTM sites were mutated. We employed the tetracycline-regulated expression (TREx) system to enable the inducible expression of exogenous RNF138 in the bulk of cells. This system was comprised of U2OS cells stably expressing the TetR tetracycline repressor (U2OS-TREx) being transfected with the pCDNA4-TO-hygromycin-superfolder green fluorescent protein (sfGFP)-MAP vector (58). The vector contains Tet operator sequences that are bound by TetR, enabling transcriptional repression of the encoded transgene in the absence of tetracycline or doxycycline (Dox), but inducible expression in their presence. Using this vector, we generated U2OS-TREx cells stably expressing siRNA-resistant RNF138-WT or its mutants tagged with all four of the FLAG, 8XHis, streptavidin binding peptide (SBP), and sfGFP tags (58). When induced with Dox, these cell lines produced fusion proteins of ∼65 kDa (Fig. 5*G*), from here on referred to as sfGFP-RNF138 constructs. The WT and K158R variants of RNF138 were expressed at similar levels as measured by quantitative immunoblotting (Figs. 5*G* and S3*G*). On the other hand, the T27A, T27E, S124A, and S124E mutants were expressed, on average, 2.7 to 3.7 times higher than WT. We attempted to adjust the Dox concentrations to induce similar expression levels of the variants, but these differences could not be eliminated at the concentrations tested (0.1 μg/ml–5 μg/ml) (Fig. S3*H*). Perhaps the differences in expression reflect other factors among the cell lines, such as the frequency of vector integration or integration in heterochromatic *versus* euchromatic regions. Nonetheless, we achieved inducible expression of exogenous RNF138 in U2OS-TREx cells, and importantly the expression levels of WT/K158R, T27A/T27E, and S124A/S124E were comparable within each pair, respectively.

**Figure 6:**
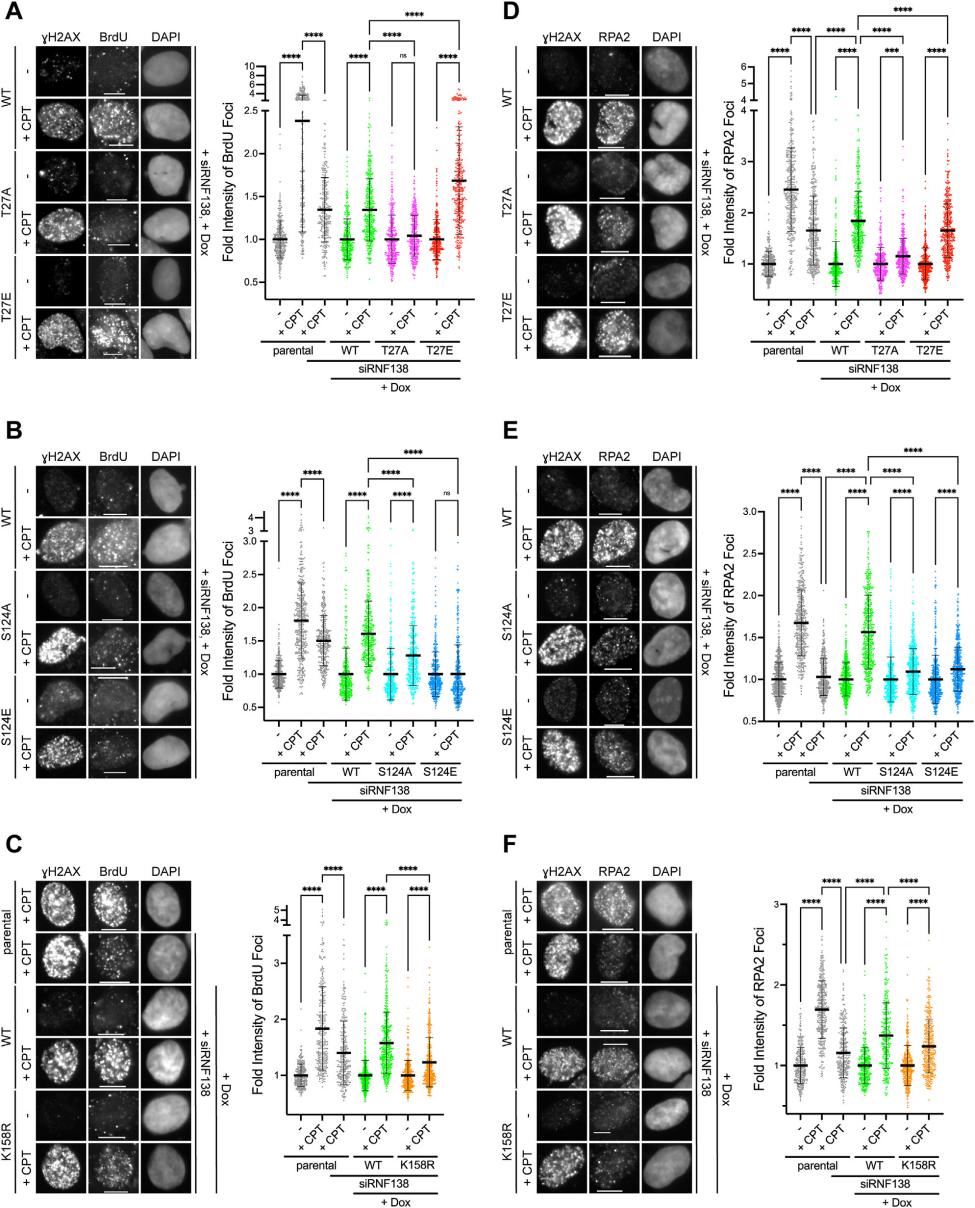
**The RNF138 PTM Sites T27, S124 and K158 are Important for DNA End Resection.***A–C*, *left panel*: Representative BrdU immunofluorescence (IF) micrographs of sfGFP-RNF138 variant-expressing U2OS-TREx cells transfected with siRNA to RNF138 (siRNF138) and treated with doxycycline (Dox) to induce expression of sfGFP-RNF138, from a single biological replicate. *C*, includes parental U2OS-TREx cells transfected with or without siRNF138. In all, cells were treated with CPT or not for 1 h. ƔH2AX was used to indicate DNA damage, while DAPI stain labeled the nucleus. *Right panel*: quantifications of relative intensity of nuclear BrdU foci. For CPT-treated cells, only ƔH2AX^+^ cells were quantified. The fluorescence intensities of foci for conditions within a given cell line were normalized to each other, with the average fluorescence intensity set at 1 for the untreated sample (no CPT). Averages were derived from the focal intensity of at least 150 cells each from two biological replicates (at least 300 cells total per condition). *D*–*F*, as in (*A*–*C*), but performing IF for RPA2 foci. Scale bars denote 10 μm. See Fig. S4, *A*–*D* for additional representative micrographs from experiments depicted in Figure 6, *A*, *B*, *D* and *E*. In all, ordinary one-way ANOVA with Šídák's multiple comparisons test was performed to determine whether differences between conditions were statistically significant. ∗∗∗∗: *p* values were <0.0001. For (*A*), ns: *p* = 0.8921. For (*B*), ns: *p* > 0.9999. For (*D*), ∗∗∗: *p* = 0.0002. BrdU, 5-bromo-2’-deoxyuridine; CPT, camptothecin; PTM, post-translational modification; RPA, replication protein A; sfGFP, superfolder green fluorescent protein; siRNA, short interfering RNA; TREx, tetracycline-regulated expression.

With the T-Rex system ready, we examined the ability for U2OS-TREx cells expressing these mutants to undergo end resection in response to treatment with CPT. CPT was chosen as it inhibits DNA Topoisomerase I (TOP1), stabilizing TOP1 cleavage complexes that, upon collision with a DNA replication fork, are converted into single-ended DSBs (59). In this way, CPT-induced DSBs are generated in an S phase-dependent manner (59), biasing repair pathway choice to DNA end resection and HR. To monitor end resection, we detected the occurrence of ssDNA by immunofluorescence staining (IF). U2OS-TREx cells were labeled with the brominated nucleoside 5-bromo-2′-deoxyuridine (BrdU), which is incorporated into DNA. The process of resection generates ssDNA, exposing an epitope on BrdU that can be detected by IF under non-denaturing conditions. The appearance of BrdU foci in response to CPT is thus indicative of active DNA end resection (60, 61). As expected, treating parental U2OS-TREx cells with CPT significantly increased the intensity of BrdU foci (Figs. 6, *A*–*C* and S4, *A* and *B*). In accordance with RNF138 being shown to promote DNA end resection (14, 20), this intensity was significantly reduced when the cells were transfected with siRNA targeting RNF138 (Figs. 6, *A*–*C* and S4, *A* and *B*). We next performed flow cytometric analysis to check if the difference was due to changes in the ratio of S phase cells. Depleting RNF138 in U2OS-TREx did not substantially alter the proportion of cells in S phase (Fig. S4*E*). Therefore, the change in BrdU focal intensity did not arise from a shift in the frequency of S phase-cycling cells, rather reflecting a perturbation in efficient DNA end resection.

We next examined the impact of RNF138 mutations on DNA end resection. U2OS-TREx cells were depleted of endogenous RNF138 and complemented with WT or mutated sfGFP-RNF138 by treatment with Dox. In agreement with T27 being an important phosphorylation site, average BrdU focal intensity in the cell line expressing the T27A mutant was substantially reduced compared to cells expressing WT-RNF138, and at levels barely above that of the same cells not treated with CPT (Fig. 6*A*), indicating dramatically reduced end resection. The contrary was observed for the T27E mutant, with mean BrdU focal intensity extensively higher than WT (Fig. 6*A*). Perhaps the negatively charged glutamate substitution results in constitutively active RNF138, constantly promoting resection. Hence, the phosphorylation of RNF138 at T27 plays a crucial role in RNF138’s ability to promote DNA end resection. Performing the same assay on the S124A mutant showed reduced BrdU focal intensity relative to WT (Fig. 6*B*), demonstrating a role for S124 phosphorylation in RNF138 function. Interestingly, unlike T27E, the S124E mutation was unable to restore BrdU focal intensity (Fig. 6*B*). Perhaps glutamate substitution at S124 is insufficient to cause the same conformational changes elicited by serine phosphorylation. Nevertheless, the fact that both S124A and S124E mutations reduce BrdU intensity underscores the importance of the S124 site in RNF138 function. We also performed the same assay for the K158R substitution mutant. U2OS-TREx cells expressing sfGFP-RNF138-K158R exhibited reduced intensity in BrdU foci relative to cells expressing WT (Fig. 6*C*), indicating K158 plays a role in RNF138’s ability to promote DNA end resection. As a control, we confirmed that concurrently depleting endogenous RNF138 while inducing expression of sfGFP-RNF138-WT with Dox did not severely alter the proportion of cells in S phase (Fig. S4*E*). We also checked if this proportion was affected by expressing any of the RNF138 mutants. The S phase fraction was similar (∼31–37%) among all the U2OS-TREx cell lines, whether expression of the sfGFP-RNF138 mutants was induced or not (Fig. S4*F*). Thus, the observed differences in BrdU focal intensity were not from cells being more or less responsive to CPT from changes in the fraction in S phase.

As active end resection generates ssDNA overhangs that are rapidly coated and protected by the binding of RPA complexes (5, 61, 62, 63), we asked if the observed changes in BrdU focal intensity would correlate with RPA binding. To do so, we performed a similar IF assay in U2OS-TREx, this time detecting extraction-resistant foci of the RPA complex subunit RPA2 in response to CPT. As seen with BrdU foci (Figs. 6, *A*–*C* and S4, *A* and *B*), knocking down RNF138 reduced RPA focal intensity (Figs. 6, *D*–*F* and S4, *C* and *D*), indicating the inhibition of end resection. Notably, CPT-induced RPA focal intensity was partially rescued upon concurrent expression of sfGFP-RNF138-WT (Fig. 6, *D*–*F*). As well, the T27A, S124A, and K158R variants exhibited significantly reduced RPA2 focal intensity relative to WT-RNF138 (Fig. 6, *D*–*F*). Likewise, RPA2 focal intensity was rescued by the T27E mutant, but not by the S124E mutant (Fig. 6*D*). Our results with RPA2 foci thereby recapitulate our findings seen with native BrdU foci.

**Figure 7:**
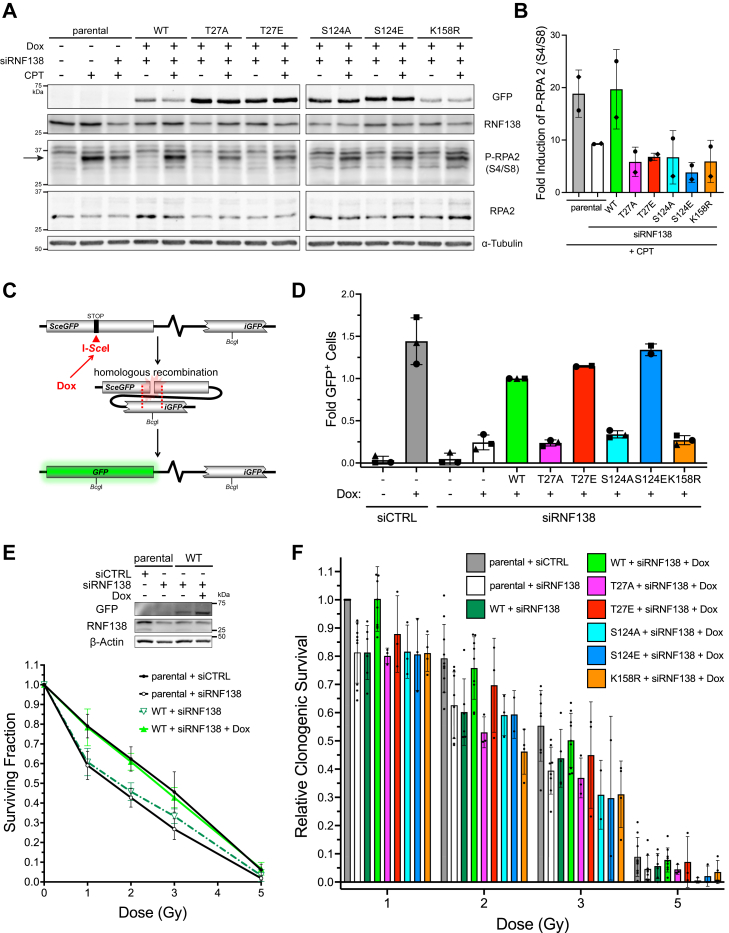
**T27, S124, and K158 on RNF138 areimportant for RPA2 Phosphorylation, HRactivity, andcellsurvival.***A*, immunoblot of whole cell extracts of parental or sfGFP-RNF138 variant-expressing U2OS-TREx cells transfected with or without siRNA to RNF138 and treated with CPT or not for 1 h. Arrow: main band for P-RPA (S4/S8); note its reduced electrophoretic mobility compared to unmodified RPA2. *B*, quantification of fold induction of P-RPA2 (S4/S8) signal from (*A*). P-RPA2 (S4/S8) signal was normalized to that of RPA2 expression. The resulting values from CPT-treated samples were then ratioed to those left untreated. *C*, schematic diagram of the DR-GFP reporter assay in TRI-DR-U2OS cells expressing doxycycline (Dox)-inducible I-*Sce*I. *D*, DR-GFP reporter assay in TRI-DR-U2OS cells transfected with siRNA to luciferase (siCTRL) or RNF138 (siRNF138) and complemented with siRNA-resistant mCherry-RNF138 variants or not. GFP^+^ cells were quantified by flow cytometry. *E*, *bottom*: clonogenic survival assay for parental or sfGFP-RNF138-WT-expressing U2OS-TREx cells transfected with siCTRL or siRNF138, induced with Dox if necessary, and treated with increasing doses of ionizing radiation. *Top*: immunoblot of endogenous and exogenous RNF138 expression of cells used for the clonogenic survival assay, at the time of irradiation. *F*, clonogenic survival assay for parental or sfGFP-RNF138 variant-expressing U2OS-TREx cells transfected with siCTRL or siRNF138, induced with Dox or not, and treated with increasing doses of ionizing radiation. Within each biological replicate, the surviving fractions obtained were normalized to the surviving fraction of the parental cells + siCTRL + 1 Gy condition. (*A*) is a representative result from at least two biological replicates. Averages were calculated from 2 (*B*), at least 2 (*D* and *F*), and 6 to 10 (*E*) biological replicates pooled together. CPT, camptothecin; RPA, replication protein A; sfGFP, superfolder green fluorescent protein; siRNA, short interfering RNA; TREx, tetracycline-regulated expression; WT, wildtype.

The N-terminal region of RPA2 is phosphorylated by the PIKKs in response to DNA damage (64). As Ser4 and Ser8 phosphorylation on RPA2 (P-S4/S8-RPA2) is dependent on CtIP (8, 20) and occurs after the binding of RPA complexes to ssDNA (64, 65, 66), it has been used as a readout for end resection (20, 40). To further support that end resection is regulated by PTMs on RNF138, we detected CPT-induced P-S4/S8-RPA2 in the same cell lines by immunoblot (Fig. 7, *A* and *B*). Consistent with our previous findings (14), cells transfected with siRNA to RNF138 exhibited reduced RPA2 phosphorylation upon stimulation with CPT. Cells knocked down of RNF138 and expressing sfGFP-RNF138-T27A, -S124A, -S124E, or -K158R could not restore P-S4/S8-RPA2 to the levels seen with WT-RNF138, agreeing with our IF data with BrdU and RPA2 foci. In fact, the cells exhibited P-S4/S8-RPA2 levels below that of parental U2OS-TREx cells treated with RNF138-targeting siRNA. Unlike the IF data however, the T27E mutant did not recover P-S4/S8-RPA2 to levels near or above WT-RNF138 (Figs. 6, *A* and *D* and 7, *A* and *B*). It may be that the resection induced by T27E, seen by IF, somehow cannot trigger RPA2 phosphorylation as effectively as having a phosphoryl group on T27, and that RPA2 phosphorylation requires events additional to RPA binding ssDNA. Still, this data reveal the importance of the T27 site in early RNF138-dependent DSB signaling. Overall, the analysis of BrdU foci, RPA2 foci, and phospho-RPA2 levels suggest phosphorylation at T27 and S124 and ubiquitylation at K158 are important for RNF138’s role in promoting DNA end resection.

### T27, S124, and K158 on RNF138 are important for HR and cell survival

In HR, the processes of homology search, strand invasion, and DNA synthesis occur downstream of DNA end resection (2). As T27, S124, and K158 affected the efficiency of end resection, we next determined if the frequency of HR was impacted by the same mutations. To do so, we used the direct repeat GFP (DR-GFP) reporter assay (67), utilizing U2OS cells integrated with two repeats of the GFP open reading frame (ORF) (Fig. 7*C*). These copies are mutated such that neither produces a fluorescent protein product; the upstream copy contains stop codons and a recognition site for the restriction endonuclease I-*Sce*I, while the downstream copy is truncated, with the N- and C-terminal regions removed. Expression of I-*Sce*I induces a site-specific DSB in the upstream copy of the GFP ORF. If the DSB is repaired by HR using the downstream copy as a template, the sequence encoding intact, fluorescent GFP is generated. Consequently, the proportion of GFP^+^ cells after I-*Sce*I expression reflects the efficiency of HR. We generated siRNA-resistant, mCherry-tagged constructs of WT-RNF138 and its PTM site mutants (T27A, T27E, S124A, S124E, K158R), then transfected them into TRI-DR-U2OS cells stably expressing Dox-inducible I-*Sce*I and depleted of endogenous RNF138. As expected, I-*Sce*I induction alone markedly increased the frequency of GFP^+^ cells, and this frequency was sharply inhibited when the cells were depleted of RNF138 (14) (Fig. 7*D*). Consistent with our IF data for end resection detected by RPA2 foci (Figs. 6, *D*–*F*, S4, *C* and *D*), adding back mCherry-RNF138-WT and -T27E in the presence of RNF138 siRNA partially rescued the frequency of HR to levels seen in cells expressing endogenous RNF138 (Fig. 7*D*). On the other hand, cells expressing the -T27A, -S124A, and -K158R mutants exhibited severely reduced HR frequencies (Fig. 7*D*). Interestingly, the S124E mutant was fully capable of restoring HR frequency, in fact to levels surpassing that of WT-RNF138, similar to the T27E mutant (Fig. 7*D*). It appears that in this specific context (DR-GFP reporter assay, mCherry-tagged RNF138), S124E resembles the actions of constitutively phosphorylated RNF138. Thus, the T27, S124, and K158 sites are important for RNF138’s ability to promote HR.

With end resection and the occurrence of HR dependent on the aforementioned PTM sites, we asked if cell survival upon DNA damage would also be impacted by mutations at the sites. To do so, we performed clonogenic survival assays on U2OS-TREx cells treated with 1 to 5 Gy of IR. Knocking down RNF138 impeded the colony-forming ability of parental U2OS-TREx cells (Fig. 7*E*), in line with previous observations (14, 20). This was not due to indirect effects on the cell cycle from RNF138 depletion, as the proportion of cells in S or G2 phase (and therefore conducive to HR) increased slightly when RNF138 was depleted (Fig. S4*B*). Conversely, clonogenic survival was rescued in sfGFP-RNF138-WT-expressing U2OS-TREx cells depleted of endogenous RNF138 (Fig. 7*E*). As a control, when Dox was not added to the culture medium, preventing expression of sfGFP-RNF138-WT, survival was reduced (Fig. 7*E*). Again, these effects were not from indirect changes on the cell cycle, as the S/G2 fraction of cells was similar whether Dox was added or not (Fig. S4*E*). Having confirmed that survival to IR is dependent on RNF138, we tested how the PTM site mutants would fare in colony forming ability using U2OS-TREx cells stably expressing the sfGFP-RNF138 variants. Consistent with our IF results for resection (Fig. 6, *A*–*F*), cells expressing the T27A, S124A, S124E, and K158R mutants exhibited relative surviving fractions similar to or below that of parental U2OS-TREx cells depleted of RNF138 (Fig. 7*F*). At 1, 2, and 3 Gy, cells expressing the T27E mutant showed intermediate survival, greater than the former mutants but less than WT-RNF138. None of these effects were from fluctuations in the cell cycle distribution, as the stable cell lines did not show appreciable differences in the S/G2 phase fraction (∼58% to 63.6%) upon Dox induction (Fig. S4*F*).

All in all, these data exemplify the role of the T27, S124, and K158R residues in promoting HR and cellular survival in the face of DSBs. They are also mostly concordant with the phenotypes observed for the mutants in DNA end resection, assayed by RPA2 and BrdU foci formation (Figs. 6, *A*–*F*, S4, *A*–*D*).

### Investigating the interplay between post-translational modifications on RNF138

**Figure 8:**
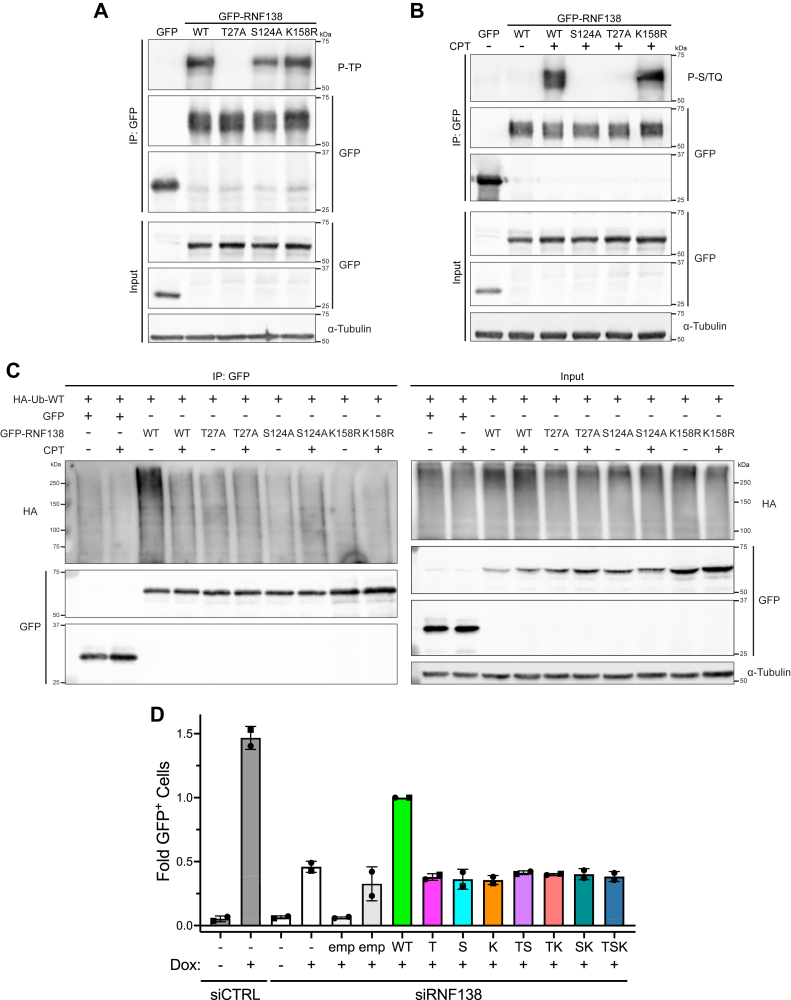
**Investigating the Interplay Between Post-Translational Modifications on T27, S124, and K158R on RNF138.***A* and *B*, stringent GFP IP were performed for HEK293 cells expressing GFP or GFP-RNF138 variants and immunoblotted (IB'd) for phosphorylated TP sites (P-TP) (*A*) or treated with CPT or not for 1 h and IB'd for phosphorylated S/TQ sites (P-S/TQ) (*B*). *C*, stringent GFP IP and subsequent IB were performed for HEK293 cells co-expressing HA-Ub-WT and GFP or GFP-RNF138 variants and treated with CPT or not for 1 h. In all samples, 10 μM MG132 was added to the culture medium starting 1 h prior to CPT treatment to improve the preservation of HA-Ub signals. *D*, DR-GFP reporter assay in TRI-DR-U2OS cells transfected with siRNA to luciferase (siCTRL) or RNF138 (siRNF138) and complemented with mCherry empty vector (emp) or siRNA-resistant mCherry-RNF138 variants or not. I-*Sce*I expression was induced by Dox. GFP^+^ cells were quantified by flow cytometry. T: T27A, S: S124A, K: K158R, TS: T27A-S124A, TK: T27A-K158R, SK: S124A-K158R, TSK: T27A-S124A-K158R. Shown are representative results from 2 (*A* and *B*) and 3 (*C*) biological replicates. Averages in (*D*) were calculated from two biological replicates pooled together. CPT, camptothecin; Dox, doxycycline; HA-Ub, HA-tagged ubiquitin; IB, immunoblotting; IP, immunoprecipitation; siRNA, short interfering RNA; WT, wildtype.

Finally, we investigated whether interplay occurs between the PTMs on RNF138. We first performed immunoprecipitation experiments on the single PTM site substitution variants of GFP-RNF138 expressed in HEK293 cells. We assessed the impact of S124A and K158R on the P-TP signal (Fig. 8*A*), the impact of T27A and K158R on the CPT-induced P-S/TQ signal (Fig. 8*B*), and the impact of T27A and S124A on RNF138 ubiquitylation by HA-Ub-WT (Fig. 8*C*). We observed that the K158R substitution had no effect on the P-TP and P-S/TQ signals on RNF138 (Fig. 8, *A* and *B*), concluding that K158 is dispensable for phosphorylation events at T27 and S124. Next, the S124A substitution partially reduced the P-TP signal (Fig. 8*A*), suggesting S124 phosphorylation may support but is not required for phosphorylation on T27. However, the T27A mutation strongly impaired the P-S/TQ signal (Fig. 8*B*). As well, basal higher-order ubiquitylation on GFP-RNF138 was notably reduced by the T27A and S124A mutations, preventing the decrease in RNF138 ubiquitylation induced by CPT from being seen (Fig. 8*C* and 3, *D* and *E*). Therefore, T27 phosphorylation may serve as a prerequisite for S124 phosphorylation, and functional T27 and S124 sites seem to be important for RNF138 ubiquitylation.

With this interdependency in mind, we asked what effect expressing the T27A-S124A (TS), T27A-K158R (TK), and S124A-K158R (SK) double mutants, as well as the T27A-S124A-K158R (TSK) triple mutant, would have on the ability for cells to perform HR. The aforementioned mutations were incorporated in siRNA-resistant mCherry-RNF138 by sequential site-directed mutagenesis, the products of which were used in the DR-GFP HR reporter assay upon concurrent depletion of endogenous RNF138. Expressing the double and triple mutants resulted in similar low HR frequencies upon I-*Sce*I expression (Fig. 8*D*). These values were also similar to those for the T27A, S124A, and K158R single mutants, which were already low to start and similar to HR frequencies for cells depleted of endogenous RNF138. As ablation of any of the PTM sites, or any combination of them, was sufficient to impair HR to baseline levels, this finding emphasizes the importance of all three sites in the function of RNF138 and suggests they could all act within the same pathway on RNF138. This is in agreement with the dependence of RNF138 ubiquitylation on the T27 and S124 residues (Fig. 8*C*) and the dependence of S124 phosphorylation on the T27 site (Fig. 8*B*).

## Discussion

Multiple reports have implicated a role for RNF138 in the DNA damage response (14, 20, 21, 22, 68, 69). As an E3 ubiquitin ligase, RNF138 facilitates the ubiquitylation of Ku80, CtIP, and Rad51D, actions that promote the occurrence of HR (14, 20, 21, 22). Little however is known about how its activity is regulated in the context of DSB repair. While RNF138 was reported to be phosphorylated by ATM in response to IR at position S124 (22), the functional significance of this modification remained unclear, as it did not affect RNF138’s recruitment to sites of DNA damage (22). Furthermore, as RNF138 stimulates Ku80 ubiquitylation in S/G2, but not G1 phase (14), it appeared RNF138 activity could be regulated by the cell cycle, although how this occurred was not previously explored. Thus, we sought to elucidate additional mechanisms by which RNF138 could be regulated. In HeLa cells, we detected a slight increase in RNF138 protein levels during the G2 phase (Fig. 1*C*), consistent with the reported increase in RNF138 messenger RNA transcripts in G2 (70). However, RNF138 protein was expressed throughout the cell cycle, and at relatively constant levels (Fig. 1, *A*–*C*). As HR is specific to the S/G2 phases (13), we speculate RNF138 expression in G1 may serve its roles outside of HR, such as binding interactors and ubiquitylating substrates in NFκB or Wnt-β-catenin signaling (23, 56, 71, 72). Regardless, it became clear that factors beyond protein level regulate RNF138 activity in HR. We located a putative CDK substrate consensus motif within RNF138’s amino acid sequence (Fig. 1*D*). In addition, RNF138 was a hit in proteomic screens for ubiquitylated proteins (52, 53, 54, 55), and ubiquitylation events are well known to govern the response to DSBs (19). We therefore addressed if ubiquitylation and CDK-dependent phosphorylation occurred on RNF138 and were involved in its function in HR.

We report here that RNF138 is phosphorylated in a CDK-dependent manner. This phosphorylation peaked during S phase, was dependent on CDK1 and CDK2, occurred on residue T27 (Figs. 1, *G*–*I* and *K*–*L* and S3*D*), and, importantly, stimulated RNF138-mediated DNA end resection (Figs. 6, *A* and *D* and 7, *A* and *B*). While the precise mechanism by which CDK phosphorylation activates RNF138 requires further study, it aligns with a pro-resection function for CDK activity in S/G2. RNF138 has been shown to mediate ubiquitylation of the pro-resection factor CtIP, enabling CtIP’s recruitment to DSBs (20). RNF138-dependent ubiquitylation of Ku80 also promotes the eviction of the DNA end-protecting and NHEJ-promoting (11, 12) Ku heterodimer from chromatin (14). As Ku displacement is required for DNA end resection to begin (15, 16), this contributes a second mode to trigger end resection. Notably, CtIP itself is phosphorylated by CDK activity, the phosphorylated version activating the endonuclease activity of Mre11 to initiate DNA end resection (6, 7, 39, 40, 41, 73). The Ku heterodimer is also a substrate for CDK activity; Ku70 is phosphorylated by CDK1 and CDK2 during S, G2, and M phase (74), and in budding yeast, CDK-phosphorylated Yku80 promotes HR and impairs NHEJ (75). Phosphorylated Ku, similar to ubiquitylated Ku (76), has been found to dissociate from DNA (15, 74). The evidence points to a model where concerted CDK activity in S and G2, acting on multiple fronts, like RNF138, CtIP, Ku, and other resection factors (36, 37, 38, 42, 43), culminates to drive the process of DNA end resection. Interestingly, our laser microirradiation data also suggests that beyond promoting DNA end resection, T27 phosphorylation may serve a minor role in regulating RNF138’s retention on chromatin (Fig. 5, *D* and *E*).

We also find that RNF138 is constitutively polyubiquitylated, with K158 serving as a site of modification (Figure 2, Figure 4, *A*–*D*, 4*B* and 8*C*). This is in line with a recent report identifying K158 as a ubiquitylation site on RNF138, with the K158R substitution able to suppress negative regulation of oncogenic MyD88 signaling (56). As the K158R mutant could not completely eliminate HA-Ub-L73P signal in GFP-RNF138 immunoprecipitates (Fig. 4*B*), there are likely other residues by which RNF138 is ubiquitylated. Indeed, four additional lysine residues on RNF138 were detected to be ubiquitylated in one proteomic screen (54). Yet, K158 is probably an abundant site for ubiquitylation, as it was the only ubiquitylation site predicted by UbPred software (56), and was consistently ubiquitylated in four proteomic screens, detected in three of these as the only site of modification on RNF138 (52, 53, 54, 55). Phenotypically, despite exhibiting reduced turnover and prolonged stability, the K158R mutant was still clearly defective in promoting DNA end resection (Figure 4, Figure 6, *C* and *D*, 6, C and F and 7, *A* and *B*). Although K158 was not essential for RNF138 recruitment to laser stripes, the K158R mutant also showed a minor impairment in recruitment (Fig. 5*C*), and this change could contribute at least partly to the defect in end resection. Overall, the data suggests ubiquitylation on K158 activates RNF138 for end resection. This is supported by the increase in ubiquitylated RNF138 in the S and G2 phases (Fig. 3, *B* and *C*), overlapping with the times end resection and HR are active. As RNF138 is appreciably expressed during all cell cycle phases (Fig. 1*C*), we suspect the enhanced ubiquitylation in S/G2 serves more to activate the protein for end resection and HR rather than actively target it for proteasomal degradation. Further study, particularly of the linkages in the ubiquitin chains conjugated to RNF138, is required to validate this idea. We also observed that RNF138 ubiquitylation was reduced upon DSBs, UV, and replication stress (Figs. 3, *D* and *E* and 8*C*). In support of this, a proteomic screen found ubiquitylation at K158 was reduced 3 h after HEK293 cells were exposed to UV irradiation (52). Since K158 ubiquitylation promotes DNA end resection (Figs. 6, *C* and *F* and 7, *A* and *B*), the reduction in ubiquitylation, seen after 1 h of genotoxic stress (Fig. 3, *D* and *E*), may reflect a negative regulatory mechanism, constraining RNF138 activity after actions to resolve the stress have commenced. For HR, this might prevent overstimulation of DNA end resection, which could lead to a loss of genetic information. Uncontrolled resection might also deplete local RPA pools, causing aberrant annealing, secondary structures, and degradation in unprotected ssDNA (63). Together, our data suggest RNF138 is regulated by ubiquitylation, with ubiquitin conjugation to K158 serving to activate the protein in DSB repair.

Our work thus identifies two additional PTMs that contribute to RNF138’s function in DSB repair, the aforementioned K158 ubiquitylation and CDK-dependent T27 phosphorylation. Both promote RNF138’s role in stimulating DNA end resection, as the T27A and K158R variants inhibit RNF138-dependent resection in response to CPT (Figs. 6, *A*, *C*, *D* and *F* and 7, *A* and *B*). Using the S124A mutant, we also demonstrate S124 phosphorylation positively regulates RNF138’s role in end resection (Fig. 6, *B* and *E* and 7, *A* and *B*), providing functional significance to the known ATM-dependent modification (22). Aligning with these observations, the T27A, S124A, and K158R mutations dramatically reduced HR frequency *in vivo* (Fig. 7*D*) and sensitized cells to DNA damage by IR (Fig. 7*F*), suggesting the defects in end resection translated to negative consequences downstream. Supporting the importance of T27 phosphorylation, the phospho-mimicking T27E variant could restore DNA end resection, HR, and clonogenic survival (Figs. 6, *A* and *D* and 7, *D* and *F*), despite having a partial defect in retention on chromatin (Fig. 5, *D* and *E*). Intriguingly, like T27E, the S124E mutant was also capable of restoring HR (Fig. 7*D*), although it was unable to rescue end resection and clonogenic survival (Fig. 6, *B* and *E* and 7, *A*, *B* and *F*). We note that the end resection and survival assays were conducted with sfGFP-RNF138, while the HR reporter assay utilized the mCherry-RNF138 fusion. Beyond the different fluorescent proteins, the sfGFP construct also contains three other epitope tags (58). We speculate construct-specific conformational differences could explain the discrepancy in results for S124E. Alternatively, it could be that the S124E variant does not sufficiently promote end resection yet is capable of activating HR through a separate mechanism. Indeed, we did not assess if the stability or recruitment of Rad51D, another target of RNF138 ubiquitylation in HR (21, 22), were impacted by the PTM site mutations in RNF138. We thus cannot conclude if the effects on HR and survival arise solely from changes in end resection or if alterations in Rad51D function also contribute. Nevertheless, our functional readouts reiterate that PTMs at T27, S124, and K158 are important to the role of RNF138 in protecting cells from DSBs.

Our data ultimately point to a scenario where phosphorylations at T27 and S124 and ubiquitylation at K158 all positively regulate RNF138 activity in end resection and HR. This may enable RNF138 to integrate signals of both DNA damage and cell cycle stage, ensuring it is fully active when DSB breaks occur during S/G2 phase. An attractive idea is that cell cycle-dependent ubiquitylation and CDK-dependent T27 phosphorylation prime RNF138 to function in S/G2. ATM-dependent phosphorylation at S124, triggered by DSBs, could perhaps give the final go-ahead signal to license RNF138 activity. Support for this notion may come from the observation that a functional T27 site is important for the DNA damage-dependent phosphorylation of RNF138 on S124 (Fig. 8*B*), suggesting T27 phosphorylation could precede phosphorylation on S124. This of course needs to be tested further, and more study is needed to decipher the molecular mechanisms by which each of the PTMs activate RNF138. In the end, our data provide additional intricacies to the tightly orchestrated molecular events triggered upon DSB damage.

## Experimental procedures

### DNA constructs and siRNAs

The FLAG-RNF138 vector (containing the full-length RNF138 ORF and a single FLAG tag (DYKDDDDK) directly C-terminal to it, within the AbVec2.0 expression vector) was a gift from Michael Hendzel (University of Alberta). pEGFP-RNF138-WT and -ΔUIM (with the RNF138 ORF C-terminal of the GFP tag) were generated previously (14); the ΔUIM mutant contains residues 1 to 228 of full-length RNF138. pCDNA3-HA-ubiquitin-WT and -L73P (both containing residues 2–76 of ubiquitin) plasmids were gifts from Tony T. Huang (New York University School of Medicine) (51). pcDNA4-TO-hygromycin-sfGFP-MAP was a gift from Dannel McCollum (Addgene plasmid #44100; http://n2t.net/addgene:44100; RRID:Addgene_44100) (58). The siRNA-resistant RNF138 ORF was inserted into the pcDNA4-TO-hygromycin-sfGFP-MAP vector between the sfGFP-N175 and His_8_ modules by GenScript Biotech, producing the pcDNA4-TO-hygromycin-sfGFP-RNF138-MAP (abbreviated sfGFP-RNF138) construct. The siRNA-resistant RNF138 ORF was inserted into the pmCherry-C1 vector by Biomatik Corporation (Kitchener, Ontario, Canada), producing the mCherry-RNF138 construct. The sfGFP-RNF138-T27A, -S124A, and -K158R mutants were generated by GenScript Biotech. All other mutants were generated using the Q5 Site-Directed Mutagenesis Kit (New England Biolabs) according to the manufacturer’s instructions. Sequences were verified by Sanger sequencing performed by the Molecular Biology Service Unit (Dept. of Biological Sciences, University of Alberta). DNA primers used for Sanger sequencing (Table S1) and site-directed mutagenesis (Table S2) and siRNA (Table S3) were custom synthesized by Sigma-Aldrich.

### Cell lines, tissue culture, and transfection of nucleic acids

All cells were maintained at 37 °C in a humidified atmosphere containing 5% CO_2_. Unless indicated, all cells were cultured in low glucose Dulbecco’s Modified Eagle’s medium (DMEM) supplemented with 10% fetal bovine serum (FBS, Gibco), 50 units/ml penicillin and 50 μg/ml streptomycin (both Gibco). Cells approaching confluency were detached using trypsin-EDTA solution, 0.25% (Sigma-Aldrich) prior to subculture. All cell lines were tested for *Mycoplasma* using DAPI staining. HeLa cells were a gift from Alfred C.O. Vertegaal (Leiden University). The HeLa HB-ubiquitin cell line (50) was a gift from Peter Kaiser (University of California, Irvine). HEK293 cells were a gift from Michael Hendzel (University of Alberta). U2OS cells stably expressing Dox-inducible I-*Sce*I and the DR-GFP reporter (TRI-DR-U2OS) were a gift from Philipp Oberdoerffer (Johns Hopkins University) (77). U2OS cells stably integrated with FRT (flippase recognition target) sites and the TetR tetracycline repressor-expressing pcDNA6/TR vector (U2OS-TREx cells) were a gift from Armin Gamper (University of Alberta). The Flp recombinase (Flp-In) system was not exploited to generate stable cell lines in U2OS-TREx. Instead, pcDNA4-TO-hygromycin-sfGFP-RNF138-MAP constructs were stably integrated into U2OS-TREx cells upon transient transfection and selection in DMEM supplemented with 10% charcoal-stripped FBS (Sigma-Aldrich) and 200 μg/ml hygromycin B (Invitrogen). To enrich for cells expressing the sfGFP-RNF138 constructs, 5 μg/ml Dox (Sigma-Aldrich) was added to the culture medium for 20 to 24 h, then GFP^+^ cells were isolated by fluorescence-activated cell sorting (FACS). U2OS-TREx cells stably expressing RNF138 constructs were maintained in DMEM with 10% FBS, 150 to 200 μg/ml hygromycin B, and 10 μg/ml blasticidin S (Gibco). Unless indicated otherwise, plasmid DNA was transfected into cells using Effectene Transfection Reagent (Qiagen) according to the manufacturer’s instructions 18 to 24 h before assays were performed. siRNA was transfected into freshly seeded cells using Lipofectamine RNAiMax Transfection Reagent (Invitrogen) once ∼48 h before assays were to be performed. Unless indicated otherwise, siRNA (Table S3) to RNF138 was transfected at a final concentration of 60 nM, while siRNAs to CDK1 and CDK2 were transfected at a final concentration of 50 nM. Control siRNA targeting luciferase was transfected at the same final concentration as the targeted siRNA.

### Cell cycle synchronization

HeLa cells were synchronized by the double thymidine block method. Thymidine (4 mM final concentration) was added to the culture medium of asynchronous cells at ∼40% confluency for 16 to 18 h (block #1). The cells were then washed twice with room temperature sterile PBS, replaced with warmed DMEM + 10% FBS, and incubated at 37 °C (release). 7 to 8 h post-release, cells were transfected with DNA constructs, if necessary. 2 to 3 h post-transfection (or 9–11 h post-release), thymidine (4 mM final concentration) was again added to the culture medium and kept for 12 to 14 h (block #2). At this point the cells were considered synchronized to the G1/S transition. To allow synchronous progression through the cell cycle, the cells were released by two washes with ice-cold PBS followed by 37 °C incubation in warm DMEM + 10% FBS. Cells were then harvested at various timepoints post-release to enrich for specific cell cycle phases (*e.g.*, 3 h for S phase, 7 h for G2 phase, 11 h for G1 phase).

### Harvesting cells

For HEK293, the cells were dislodged from the culture vessel by flushing the surface with the culture medium. Cells were then pelleted by centrifugation at 525*g* for 5 min at 4 °C. The other cell lines were dislodged via trypsinization: they were washed twice in ice-cold PBS (137 mM NaCl, 2.7 mM KCl, 10 mM Na_2_HPO_4_, 1.8 mM KH_2_PO_4_, pH 7.4), detached with trypsin-EDTA solution, 0.25% (Sigma-Aldrich) at 37 °C for 5 min, resuspended into four volumes of ice-cold DMEM + 10% FBS, and pelleted by centrifugation at 525*g* for 5 min at 4 °C. All cells were then resuspended into ice-cold PBS and centrifuged again (525*g*, 5 min, 4 °C). After removing the supernatant, the pellet was flash frozen in liquid nitrogen before storage at −80 °C.

### Cell cycle profiling by flow cytometry

If needed, this procedure was performed during the above cell harvesting method. When cells were resuspended in PBS after the first centrifugation, 10 to 20% of the cell pellet was saved. To this fraction, much of the supernatant was removed, after which the cells were vortexed into an ice-cold mixture of PBS prepared with 70% ethanol as the solvent. The cells were fixed by −20 °C incubation for at least 30 min. The cells were washed once in PBS, then tumbled end-over-end for 30 min at room temperature in PBS containing 100 μg/ml RNase A (Invitrogen) and 3.8 mM sodium citrate. Propidium iodide was added to a final concentration of 50 μg/ml, and the cells were again tumbled end-over-end at room temperature for at least 30 min. The propidium iodide intensity was then measured for single cells by flow cytometry using a FACSCanto II (BD Biosciences).

### Cell treatments

All inhibitors were purchased from Millipore-Sigma or Selleck Chemicals and dissolved in DMSO (or, for hydroxyurea, water). Inhibitors were diluted in warmed (37 °C) culture medium immediately prior to cell treatment. Vehicle controls contained only the solvent of the inhibitors diluted to the same extent. Unless indicated otherwise, cells were treated with the following concentrations of inhibitors: 25 μM roscovitine, 2.5 μM AZD5438, 10 μM RO-3306, 10 μM SB203580. For treatment with ultraviolet light (UV), cell monolayers were washed in PBS, which was then removed, and exposed to 20 s of UV (equivalent to ∼60 J/m^2^). The culture medium was quickly re-added and cells were incubated at 37 °C for 1 h after which they were harvested. For treatment with ionizing radiation, cells were exposed to 10 Gy from a ^60^Co source (Gammacell 220 Irradiation Unit, purchased 1978, Atomic Energy of Canada Limited), allowed to recover for 1 h at 37 °C, then harvested. For the remaining DNA damaging agents, cells were replaced with culture medium containing the agents and incubated at 37 °C (1 μM camptothecin for 1 h; 25 μM phleomycin for 1 h; 2 mM hydroxyurea for 4 h), then harvested.

### Preparation of whole cell extracts

Frozen pellets (obtained from the above cell harvesting protocol) were resuspended into ice-cold High SDS Lysis Buffer (25 mM HEPES pH 7.4, 500 mM NaCl, 2% sodium dodecyl sulfate, 1% Triton X-100, 0.5% sodium deoxycholate, 1 mM EDTA) supplemented with 2× cOmplete protease inhibitor cocktail, EDTA-free (cOmplete, Roche) and 1× phosSTOP phosphatase inhibitor cocktail (phosSTOP, Roche). The mixture was then sonicated with a Fisher Scientific Model 705 Sonic Dismembrator with microtip probe (at amplitude 1–5, for 1 min). 4× SDS Sample Buffer (250 mM Tris pH 6.8, 8% sodium dodecyl sulfate, 40% glycerol, 0.2% bromophenol blue) was then added to attain a final concentration of 1×, while 2-mercaptoethanol (BME) was added to a final concentration of 5%. The samples were treated at 95 °C for 5 min at 900 rpm on a ThermoMixer F1.5 (Eppendorf) prior to resolution by SDS-PAGE.

### SDS-PAGE and immunoblotting

Samples were loaded into hand-cast mini-gels comprising Tris (37.5 mM, pH 8.8 for the resolving layer; 12.5 mM, pH 6.8 for the stacking layer), 0.1% sodium dodecyl sulfate (SDS), and 5 to 12% polyacrylamide. Precision Plus protein dual color standards (Bio-Rad) were loaded as the molecular weight ladder. Electrophoresis was performed at 150 V in Running Buffer (25 mM Tris pH 8.3, 192 mM glycine, 0.1% SDS). The resolved proteins were then wet electro-transferred onto 0.2 μm nitrocellulose membrane for 1 h at 110 V in Transfer Buffer (25 mM Tris pH 8.3, 192 mM glycine, 20% methanol). To perform immunoblot, the nitrocellulose was first blocked in 4% fish skin gelatin (FSG) dissolved in TBS (50 mM Tris pH 7.5, 150 mM NaCl) at room temperature. TBS with 5% bovine serum albumin (BSA) was used as the blocking solution when phospho-specific antibodies were used as the primary antibody. The primary antibodies were diluted in freshly prepared TBS + 0.1% Tween-20 (TBST). For phospho-specific antibodies, 5% BSA was included in this solution, while for the anti-RNF138 antibodies, 2% FSG was included. The diluted antibodies were incubated with the membranes for either 1 h at room temperature or overnight at 4 °C under gentle rocking. The membranes were then shaken in TBST (3 times, 10 min each), incubated for 1 h at room temperature with horseradish peroxidase (HRP)-, IRDye 680RD- or IRDye 800CW-conjugated secondaries (all LI-COR Biosciences) in TBST under gentle rocking, and shaken in TBST (3 times, 10 min each) and TBS (once, 10 min). HRP activity was detected by incubating the membranes in Amersham ECL Prime Western Blotting Detection Reagent (Cytiva) for 2 min. Enhanced chemiluminescence (HRP) or fluorescence (IRDye) signals were acquired on the Odyssey Fc Imaging System and quantified by densitometry with Image Studio software (both LI-COR Biosciences). If re-probing was required, membranes were treated with Stripping Buffer (100 mM glycine pH 2.2, 1% SDS) for 30 min with vigorous shaking, rinsed with distilled water, and air-dried overnight. They were then re-blocked and probed with the necessary primary and secondary antibodies. To ensure immunoblots for loading controls of whole cell extracts (actin, tubulin) and immunoprecipitations (anti-GFP on GFP-RNF138, anti-FLAG on FLAG-RNF138) could be quantified without saturation, in such situations primary antibodies were used at low concentrations (Table S4) and blots were detected via fluorescence (IRDye) instead of enhanced chemiluminescence.

### FLAG immunoprecipitation

Pellets of FLAG-RNF138-expressing HeLa cells from a 100 mm dish were resuspended into ice-cold NETN-500 (50 mM Tris pH 8.0 at 4 °C, 500 mM NaCl, 0.5% IGEPAL CA-630, 1 mM EDTA) supplemented with fresh 2× cOmplete, 1.25× phosSTOP, and 50 mM N-ethylmaleimide (NEM) and shaken on ice (250 rpm, 20 min). The lysate was clarified by centrifugation at 20,000*g* for 15 min at 4 °C, and the resulting pellet was discarded. 10% of the supernatant was saved as an input control and mixed with an equal volume of 2× SDS Sample Buffer (125 mM Tris pH 6.8, 4% sodium dodecyl sulfate, 20% glycerol, 0.1% bromophenol blue) along with BME to a final concentration of 5%. The input control was then heated to 95 °C for 5 min at 900 rpm on a ThermoMixer F1.5. The remaining supernatant (90%) was diluted to reduce the NaCl concentration to 150 mM, then mixed with 20 μl of anti-FLAG M2 magnetic beads (Sigma) that were pre-washed twice in ice-cold TBS + 0.5% IGEPAL CA-630 (TBSN). The mixture was tumbled end-over-end for 2 h at 4 °C. Non-specific binding was removed by four washes in ice-cold TBSN. Each wash involved vortexing for 20 s, centrifuging at 2700*g* for 2 min, placing the sample on a magnetic rack, and aspirating the supernatant. Bound proteins were eluted off the beads by adding 2× SDS Sample Buffer and heating for 10 min at 95 °C at 1200 rpm (ThermoMixer F1.5). The eluate and input control fractions were then processed for SDS-PAGE and immunoblotted. For the eluate fraction, the modification of interest (*e.g.* phosphorylation) was blotted for first, then the membrane was stripped and re-probed to detect immunoprecipitated FLAG-RNF138.

### GFP immunoprecipitation

Pellets of GFP construct-expressing HEK293 cells from a 100 mm dish were resuspended into ice-cold RIPA Buffer (50 mM Tris pH 7.4 at 4 °C, 150 mM NaCl, 1% IGEPAL CA-630, 1% sodium deoxycholate, 0.1% SDS, 1 mM EDTA) supplemented with fresh 2× cOmplete, 1.25× phosSTOP, and 50 mM NEM and shaken on ice (250 rpm, 20 min). The lysate was centrifuged (20,000*g*, 15 min, 4 °C), and the pellet discarded. 10% of the supernatant was taken out as an input control and processed as in the FLAG Immunoprecipitation procedure (above). The remaining supernatant (90%) was mixed with 15 μl of GFP Selector agarose beads (NanoTag Biotechnologies) that were pre-washed twice in ice-cold RIPA Buffer, then tumbled end-over-end at 4 °C for 1 h. To remove non-specific binding, the beads were washed twice in ice-cold RIPA Buffer, then 4 times in ice-cold Stringent Wash Buffer (50 mM Tris pH 8.0 at 4 °C, 2 M NaCl, 1% IGEPAL CA-630, 0.25% sodium deoxycholate, 0.1% SDS, 1 mM EDTA). Each wash involved vortexing for 20 s, centrifuging at 3000*g* for 2 min, and aspirating the supernatant. Bound proteins were eluted by adding to the beads 2× SDS Sample Buffer with 5% BME and heating on the Thermomixer (95 °C, 30 min, 1200 rpm). Both the input and eluate fractions were then subjected to SDS-PAGE and immunoblot analysis. For the eluate fraction, the modification of interest (*e.g.* ubiquitylation) was blotted for first, then the membrane was stripped and re-probed to detect the immunoprecipitated GFP-tagged protein.

### Co-immunoprecipitation

Pellets of GFP construct-expressing HEK293 cells from a 100 mm dish were processed as in the procedure for GFP Immunoprecipitation (above), with the following changes: NETN-150 (50 mM Tris pH 8.0 at 4 °C, 150 mM NaCl, 0.5% IGEPAL CA-630, 1 mM EDTA) was used in place of RIPA Buffer, 20 μl of GFP Selector agarose beads (NanoTag Biotechnologies) were used, and the beads were washed 4 times with only NETN-150 to remove non-specific interactions.

### CDK2 *in vitro* kinase assay

HEK293 cells were transfected with DNA constructs encoding GFP, GFP-RNF138-WT, or GFP-RNF138-T27A (2 150 mm dishes per construct) and harvested the next day. The cell pellets were then resuspended into ice-cold RIPA Buffer (as above) supplemented with fresh 2× cOmplete and 1× phosSTOP and shaken on ice (250 rpm, 20 min), after which the lysates were clarified by centrifugation (20,000*g*, 15 min, 4 °C) and tumbled end-over-end at 4 °C for ∼3 to 4 h with 20 μl of GFP Selector agarose beads that had been pre-washed twice in RIPA Buffer. The beads were then washed three times in ice-cold RIPA Buffer and three times in ice-cold TBSN buffer (as above). Washes were performed as per the GFP Immunoprecipitation procedure (above). Any residual TBSN above the beads was then removed, and the bead suspension for each construct was evenly split into two portions of ∼13 μl each. Each portion was promptly mixed on ice with components from a CDK2 Assay Kit (catalogue number 79599, BPS Bioscience, San Diego, CA, USA) to a final volume of 45 μl: 6 μl of 5× kinase assay buffer 1, 1 μl of 500 μM ATP, 5 μl of sterile double-distilled water, and 20 μl of either CDK2/Cyclin A2 mixture (concentration of stock: 2.5 ng/μl in 1× kinase assay buffer 1) or 1× kinase buffer 1. The reaction mixtures were then incubated at 30 °C (300 rpm, ThermoMixer F1.5). The reaction was quenched 45 min later by adding 45 μl of 2× SDS Sample Buffer and heating the mixture to 95 °C for 5 min (900 rpm, ThermoMixer F1.5). Upon SDS-PAGE, the relative kinase activity of CDK2 was detected by immunoblotting for phospho-Thr-Pro (P-TP) and GFP.

### Isolation of ubiquitin conjugates by nickel affinity purification

HeLa HB-ubiquitin cells from 150 mm dishes were harvested as described above, except prior to pelleting for flash freezing, the cells were resuspended into ice-cold PBS and 10% of each sample was taken out to serve as the input control. All samples were then pelleted by centrifugation (525*g*, 5 min, 4 °C) and flash frozen in liquid nitrogen before storage at −80 °C. The 10% input control was processed separately for lysis to prepare whole cell extract (as above) while the remainder was processed for nickel affinity purification, described here. All buffers were prepared at most 4 h before use. All washes entailed vortexing in the indicated buffer for at least 20 s, centrifugation at 750*g* for 2 min, and removal of the supernatant *via* vacuum aspiration. Cell pellets were dissociated into ice-cold Guanidine Lysis Buffer (6 M guanidine-HCl, 100 mM sodium phosphate buffer pH 8.0, 10 mM Tris, 5 mM imidazole, 5 mM BME) by vortex and sonicated for 1 min (amplitude 25, Fisher Scientific Model 705 Sonic Dismembrator with microtip probe). The lysate was then mixed with 150 μl of Ni-NTA agarose beads (Qiagen) that were pre-washed three times in Guanidine Lysis Buffer. The mixture was agitated on a rocker for 4 h at room temperature. Non-specific interactions were removed with sequential washes at room temperature: once in Guanidine Wash Buffer (6 M guanidine-HCl, 100 mM sodium phosphate buffer pH 8.0, 10 mM Tris, 10 mM imidazole, 0.1% Triton X-100, 5 mM BME), once in pH 8 Urea Wash Buffer (8 M urea, 100 mM sodium phosphate buffer pH 8.0, 10 mM Tris, 10 mM imidazole, 0.1% Triton X-100, 5 mM BME), and three times in pH 6.3 Urea Wash Buffer (8 M urea, 100 mM sodium phosphate buffer pH 6.3, 10 mM Tris, 0.1% Triton X-100, 5 mM BME). Bound proteins were eluted off the beads with Ni-NTA Elution Buffer (150 mM Tris pH 6.7, 200 mM imidazole, 5% sodium dodecyl sulfate, 30% glycerol, 0.05% bromophenol blue, 5% BME) at 60 °C for 30 min on a ThermoMixer F1.5 (Eppendorf) at 1200 rpm and resolved by SDS-PAGE for subsequent immunoblotting.

### Cycloheximide chase assay

HEK293 cells were transfected with the indicated GFP-RNF138 DNA constructs. 20 h later, cycloheximide was added to the culture medium to 100 μg/ml with or without MG132 (5 μM final concentration). Cells were incubated for various timepoints up to 16 h and then harvested. Whole cell extracts were prepared, and protein expression levels were analyzed by fluorescence-based immunoblotting, with GFP intensity normalized to the intensity of actin.

### Laser microirradiation of live cells

U2OS cells were seeded on 35 mm glass-bottom dishes (MatTek Corporation). 24 h later, they were transfected with 200 ng of the indicated DNA construct and incubated for ∼16 h. Prior to imaging, cells were pre-sensitized with 0.5 μg/ml Hoechst 33258 for 30 min at 37 °C, washed with PBS, and replaced with warmed phenol red-free DMEM containing 25 mM HEPES + 10% FBS (both Gibco). The dishes were maintained at 37 °C in a humidified 5% CO_2_ atmosphere while being imaged on a Nipkow spinning disk confocal system (UltraVIEW ERS, PerkinElmer) mounted on an Axiovert 200M inverted microscope (Zeiss) and equipped with an sCMOS camera (Prime BSI, Photometrics). Localized DNA damage was induced in a single 1 μm thick line spanning the width of the cell nucleus using a 5 mW 405 nm diode laser coupled to a FRAP (fluorescence recovery after photobleaching) module (UltraVIEW Photokinesis, PerkinElmer) with the following settings: 20% power output, 20 iterations. The GFP fusion proteins were then excited with a 488 nm argon laser and seen through a 63×, 1.4 numerical aperture oil immersion DIC Plan-Apochromat objective lens (Zeiss). Time-lapse images in the GFP emission channel were recorded using Volocity 6.3 software (PerkinElmer). The fluorescence intensity at the laser stripe over time was determined *via* Image J software. Measurements from 30 to 75 cells pooled from three independent experiments were averaged.

### Immunofluorescence staining

Stable cell lines generated in U2OS-TREx were seeded onto sterilized glass coverslips (#1.5 thickness, Electron Microscopy Sciences) and transfected with siRNA 1 h later. ∼28 h post-transfection, 5 μg/ml Dox was added to the culture medium for ∼16 to 20 h to induce sfGFP-RNF138 expression. After a 1 h treatment with 1 μM camptothecin, the cells were washed twice with ice-cold PBS, then incubated twice, 3 min each, with ice-cold RPA Extraction Buffer (25 mM HEPES pH 7.9 at 4 °C, 300 mM sucrose, 50 mM NaCl, 0.5% Triton X-100, 1 mM EDTA, 3 mM MgCl_2_). They were then washed again twice with ice-cold PBS prior to fixation at room temperature for 20 min in 2% paraformaldehyde in PBS. The reaction was quenched for 10 min in 100 mM NH_4_Cl in PBS, and the cells were permeabilized for 5 min in PBS + 0.5% Tween-20. The cells were then incubated with primary antibodies for 1 h at room temperature or overnight at 4 °C, incubated in PBS + 0.1% Tween-20 for 5 min, washed 6 times in PBS, and incubated with secondary antibodies for 1 h at room temperature. All antibodies were diluted in PBS, and antibody incubations were performed with the coverslips being inverted into 75 μl droplets of the antibody solution. The cells were then counterstained in 10 ng/μl DAPI in PBS for 20 min, washed 6 times in PBS, and mounted onto microscopy slides in Mounting Medium (2% propyl gallate in PBS prepared with 10% DMSO and 80% glycerol as the solvent). Images were acquired on a fluorescence microscope (Zeiss AxioImager.Z2) using version 7.10.4 of MetaMorph software (Molecular Devices). The microscope utilized a 1.4 numerical aperture 40× oil immersion DIC M27 Plan-Apochromat objective lens (Zeiss) and Prime BSI sCMOS camera (Teledyne Photometrics). Extraction-resistant nuclear foci were quantified using the Cell Intensity Mean of Vesicles feature of the Statistics module of Imaris x64 software version 9.9.1 (Oxford Instruments).

### *In vivo* homologous recombination (DR-GFP) reporter assay

Per condition, ∼4 × 10^6^ TRI-DR-U2OS cells were electroporated with 60 nM siRNA and if necessary, 2 μg of mCherry-RNF138 DNA via a 4D-Nucleofector X Unit (program CM-104) with SE Cell Line 4D-Nucleofector X Kit L (both Lonza Bioscience). 8 h post-transfection, 1 μg/ml Dox was added to the culture medium for 24 h to induce expression of I-*Sce*I. The culture medium was then replaced and cells were cultured without Dox for another 24 h. Cells were collected according to the above cell harvesting procedure except that instead of being flash frozen, the cells were vortexed into 2% paraformaldehyde in PBS and incubated for 20 min for fixation. The cells were then washed 3 times in PBS. The frequency of GFP^+^ cells was measured by flow cytometry (FACSCanto II, BD Biosciences) on at least 100,000 cells.

### Clonogenic survival assay

Stable cell lines of U2OS-TREx were seeded in 100 mm dishes and transfected with siRNA 1 h later. ∼40 h post-transfection, the cells were detached by trypsinization and kept on ice. The cell density was measured on an automated cell counter (Corning) equipped with CytoSMART Cloud (CytoSMART Technologies). Cells were then seeded in duplicate onto 60 mm dishes and incubated at 37 °C with or without 5 μg/ml Dox for ∼16 h. The number of cells seeded per dish were as follows: parental + siCTRL – 900 cells, parental or sfGFP-RNF138-WT + siRNF138 – 4000 cells, all other cell lines + siRNF138 – 12,000 cells. The dishes were exposed to up to 5 Gy of ionizing radiation from a ^60^Co source (Gammacell 220 Irradiation Unit, purchased 1978, Atomic Energy of Canada Limited), after which the culture medium was replaced. The cells were incubated at 37 °C for 7 to 10 days; 5 μg/ml Dox was included in the medium if sfGFP-RNF138 expression was required. The medium was then removed and cells were fixed and stained in 0.5% Crystal Violet/25% methanol. Colonies of ≥50 cells were counted. The surviving fraction was calculated by dividing the number of colonies formed at a given dose by the number that formed at 0 Gy.

### Sequence alignment

Amino acid sequences of RNF138 orthologues were obtained from UniProt, aligned with Clustal Omega (European Bioinformatics Institute, European Molecular Biology Laboratory), and annotated in ESPript 3.0 (78).

### AlphaFold modeling

RNF138 modeling and predictions were performed using the ColabFold implementation of AlphaFold (https://github.com/sokrypton/ColabFold) (79, 80, 81, 82). The AlphaFold_MMseqs2 Google Colab notebook (version 1.5.2) was used as previously described (83). Confidence metrics were plotted with Microsoft Excel (version 2204) and Morpheus (https://software.broadinstitute.org/morpheus).

### Image and data processing

Raw micrographs and immunoblot scans were adjusted with the Levels tool and cropped in Adobe Photoshop 2023 and 2021, respectively. For immunofluorescence micrographs, images from the same biological replicate were scaled to identical settings. For GFP immunoprecipitation assays, GFP-RNF138 ubiquitylation was quantified from densitometry readings of immunoblots with Image Studio software (LI-COR Biosciences). HA signal from immunoprecipitates was normalized to the GFP signal immunoprecipitated. All graphs were generated in Prism 9 (GraphPad) and display the mean with error bars showing the standard deviation. Unless indicated otherwise, ordinary one-way ANOVA with Šídák's multiple comparisons test was performed to determine statistical significance. Asterisks depict statistically significant differences: ns (not significant), ∗ (*p* ≤ 0.05), ∗∗ (*p* ≤0.01), ∗∗∗ (*p* < 0.001), ∗∗∗∗ (*p* < 0.0001). Figures were arranged and labeled using Adobe Illustrator 2023.

## Data availability

All data described herein are contained within the manuscript. This study generated a collection of plasmids, cell lines and recombinant proteins. All materials will be distributed upon request after publication.

## Supporting information

This article contains supporting information (Figs. S1, S2, S3, S4, and Tables S1, S2, S3, S4) in the Supporting information file (20, 44, 79, 80, 81, 82, 84).

## Conflict of interest

The authors declare that they have no conflicts of interest with the contents of this article.

## Supporting information

Figure S1 **Related to**Figure 1**.***A*, immunoblots (IBs) of U2OS cells were transfected with siRNA targeting luciferase (siCTRL) or the *RNF138* gene (siRNF138) within its coding or 3′ untranslated region (UTR). siRNA targeting the *RNF138* UTR was comprised of a pool of four siRNAs, each at 40 nM. *B*, results from the scan of the primary sequence of full-length human RNF138 by the GPS (Group-based Prediction System) 6.0 web server (44) for consensus phosphorylation motifs of the cyclin-dependent kinase (CDK) subset of the CMGC family of kinases (http://gps.biocuckoo.cn/online.php).

Figure S2 **AlphaFold-Predicted Model of RNF138’s Structure.***A*, predicted model (79, 81, 82) of human RNF138 (UniProt accession Q8WVD3) with individual domains coloured respectively: linkers in white, RING in green, zinc fingers (ZNF) in blue, ubiquitin interacting motif (UIM) in dark yellow. The RING domain folds onto ZNF1, linked together by a hinge-like linker. A longer linker (54 residues) connects the N-terminal RING and ZNF1 to ZNF2 and ZNF3, which appear to pack and form a folded structure and are followed by the UIM. The post-translational modification sites described in this study are highlighted: T27 (*red*), S124 (*dark blue*), and K158 (*orange*). *B*, closeup view of residue T27 in (*A*), highlighting positive residues in close proximity and forming a positively charged surface in the RING domain. *C* and *D*, Predicted Local Distance Difference Test (pLDDT) (84) plot of the predicted model of RNF138. pLDDT scores range from 0 to 100, where scores above 70 indicate a high level of confidence in the position of the Cɑ atom, and scores above 90 indicate a high level of confidence in the placement of side chain atoms. Scores below 50 are generally considered as indications of low confidence and suggest disorder (84). Dark blue and red correspond to high confidence (>90) and low confidence (<50) pLDDT scores, respectively. The colour gradient on the graph (*D*) corresponds to the same colour scheme in panel (*C*). Overall, the model was predicted with high confidence with a median pLDDT of 88.9. The residues in the N-terminal RING domain and ZNF1 had consistently high pLDDT high values. Notably, T27 had a score of 91.6. The linker connecting the N-terminal domains to the C-terminal domains was predicted with low confidence, suggesting flexibility within those residues. S124 is positioned in the flexible linker with a pLDDT score of 26.05, suggesting a dynamic or flexible region (79, 80, 81, 84). The C-terminal zinc fingers and UIM were predicted with high confidence. Interestingly, residue K158, preceding ZNF2, had a pLDDT score of 94.12. *E*, plot representing the predicted alignment error (pAE) for the predicted structure of RNF138. In contrast to pLDDT, which measures local confidence, pAE is a long-range domain position confidence metric that calculates the confidence of each residue in relation to all other residues (79, 80, 81, 82). It quantifies the predicted error in Angstroms (Å) for residue *x* if the model were aligned onto the true structure at residue *y*. The pAEs within the N-terminal domains of RNF138 were of high confidence, with a median predicted error of 2.82 Å. The flexible linker connecting the N-terminal and C-terminal domains had high predicted error, with a median pAE value of 22.36 Å, further suggesting a flexible region in the protein. The C-terminal domains had a median pAE of 5.1 Å, suggesting a high confidence prediction. pAE is practical for measuring and analyzing both intra- and interposition confidence because it does not impose the requirement for the residues to belong to the same monomer (79, 80). Although the N- and C-terminal domains are within the same monomer and appear to fold and interact, they are separated by the large flexible linker. The pAE error between the N-terminal domains and C-terminal domains is relatively high (16.77 Å) due to the dynamic linker making it difficult to confidently predict residues involved in the interaction between the N- and C-terminal domains. Overall, this modeling suggests RNF138 is a flexible and dynamic protein accessible to post-translational modification.

Figure S3 **Related to**Figure 1, Figure 2**and**5**.***A*, FLAG immunoprecipitates (IPs) of HEK293 cells expressing FLAG-RNF138 or not were immunoblotted (IB'd) for the FLAG epitope or RNF138 (Abcam antibody). *B*, G2-synchronized HeLa cells were treated with 0.2% DMSO vehicle control (veh), 50 μM roscovitine (rosco), or the MAP kinase inhibitor SB203580 (MAPKi) at 10 μM for 4 h. Cell extracts were then IP’d for RNF138 or with control immunoglobulin G (IgG) and IB'd for phosphorylated TP sites (P-TP) or RNF138. *C*, flow cytometric analysis of propidium iodide signal in HeLa cells treated as in Figure 1*I*. Averages in (*C*) were calculated from 3 biological replicates pooled together. *D*, FLAG IP from asynchronous HeLa cells expressing FLAG-RNF138-WT or -T27A and IB'd for P-TP and FLAG. The first run of the IP and the input control is shown in Figure 1*L*. The second run of the IP is shown here; one-fourth the volume of the IP eluate from the first run is loaded. mIgG: mouse anti-FLAG immunoglobulin G fragments used for IP, detected by the anti-mouse—HRP secondary antibody. *E*, 90% of a single pellet of plain HeLa cells and 3 cell pellets of HeLa stably expressing 6XHis-biotin-ubiquitin (HeLa HB-ubiquitin) was subjected to nickel affinity purification (“His pull-down”), while the remaining 10% was processed to generate whole cell extract (input). The samples were resolved by SDS-PAGE and transferred to nitrocellulose membrane. The His pull-down fraction was first stained for total protein with REVERT total protein stain (LI-COR Biosciences), then IB’d for RNF138, and finally stripped and IB’d for ubiquitin. For the remaining 10% of cells, whole cell extracts were prepared for the input control (rightmost panel) and IB’d for RNF138. Note the RNF138 antibody detects other species beyond RNF138 (∗). RNF138 itself (predicted molecular weight: 28 kDa) is detected just above 25 kDa (arrow). MW: molecular weight standards. *F*, schematic diagrams of the structural domains in wildtype (WT) and the ΔUIM mutant of GFP-tagged RNF138. *G*, quantification of IBs (such as Fig. 5*G*) for doxycycline (Dox)-inducible expression of sfGFP-RNF138 variants in U2OS-TREx cells stably integrated with their DNA. *H*, example titration of Dox concentrations to induce sfGFP-RNF138-WT and -T27E expression, detected by IB. (*D*) is a representative result from at least 6 biological replicates. Averages in (*G*) were calculated from at least 7 biological replicates pooled together.

Figure S4 **Related to**Figures 6**and**7**.***A* and *B*, additional representative micrographs from Figure 6. Representative BrdU immunofluorescence (IF) micrographs of parental U2OS-TREx cells transfected with or without siRNA to RNF138 (siRNF138) and treated with camptothecin (CPT) or not for 1 h. ƔH2AX was used to indicate DNA damage, while DAPI stain labeled the nucleus. Micrographs are derived from the same biological replicates presented in the left panels of Figure 6*A* (*A*) and Figure 6*B* (*B*), respectively. *C* and *D*, as per (*A* and *B*), but for RPA2 foci instead. Micrographs are derived from the same biological replicates presented in the left panels of Figure 6*D* (*C*) and Figure 6*E* (*D*), respectively. *E*, flow cytometric analysis of propidium iodide signal in parental or sfGFP-RNF138-WT-expressing U2OS-TREx cells transfected with siRNA to RNF138 or not, and with or without doxycycline (Dox) induction. *F*, flow cytometric analysis of propidium iodide signal in sfGFP-RNF138 variant-expressing U2OS-TREx cells induced with Dox or not. Averages were calculated from 2 (*F*) or at least 2 (*E*) biological replicates pooled together. Scale bars denote 10 μm.
