# Supplementary figures and images for: The role of RNF138 in DNA end resection is regulated by ubiquitylation and CDK phosphorylation
Source: J Biol Chem. 2024 Feb 1;300(3):105709. doi: 10.1016/j.jbc.2024.105709 (PMC10910129; doi:10.1016/j.jbc.2024.105709)

**A**

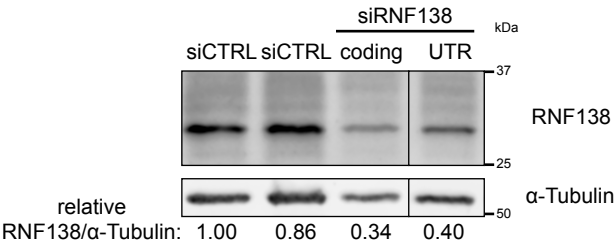

**B**

1 item:

| ID              | Position | Code | Kinase   | Peptide         | Score  | Cutoff |
|-----------------|----------|------|----------|-----------------|--------|--------|
| Q8WVD3   RNF138 | 27       | T    | CMGC/CDK | VCQEVLKTPVRTTAC | 0.1351 | 0.0164 |

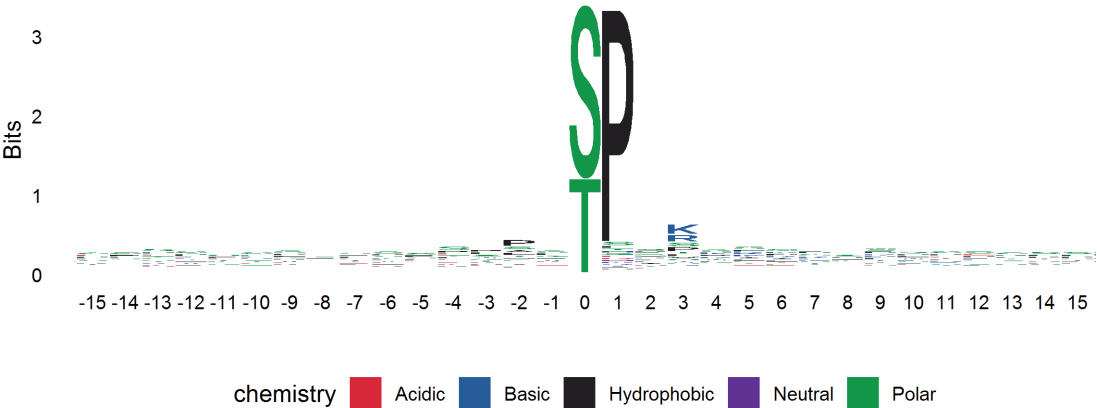

Supplement: Figure S1 — Related toFigure 1.A, immunoblots (IBs) of U2OS cells were transfected with siRNA targeting luciferase (siCTRL) or the RNF138 gene (siRNF138) within its coding or 3′ untranslated region (UTR). siRNA targeting the RNF138 UTR was comprised of a pool of four siRNAs, each at 40 nM. B, results from the scan of the primary sequence of full-length human RNF138 by the GPS (Group-based Prediction System) 6.0 web server (44) for consensus phosphorylation motifs of the cyclin-dependent kinase (CDK) subset of the CMGC family of kinases (http://gps.biocuckoo.cn/online.php). [file mmc1.pdf]

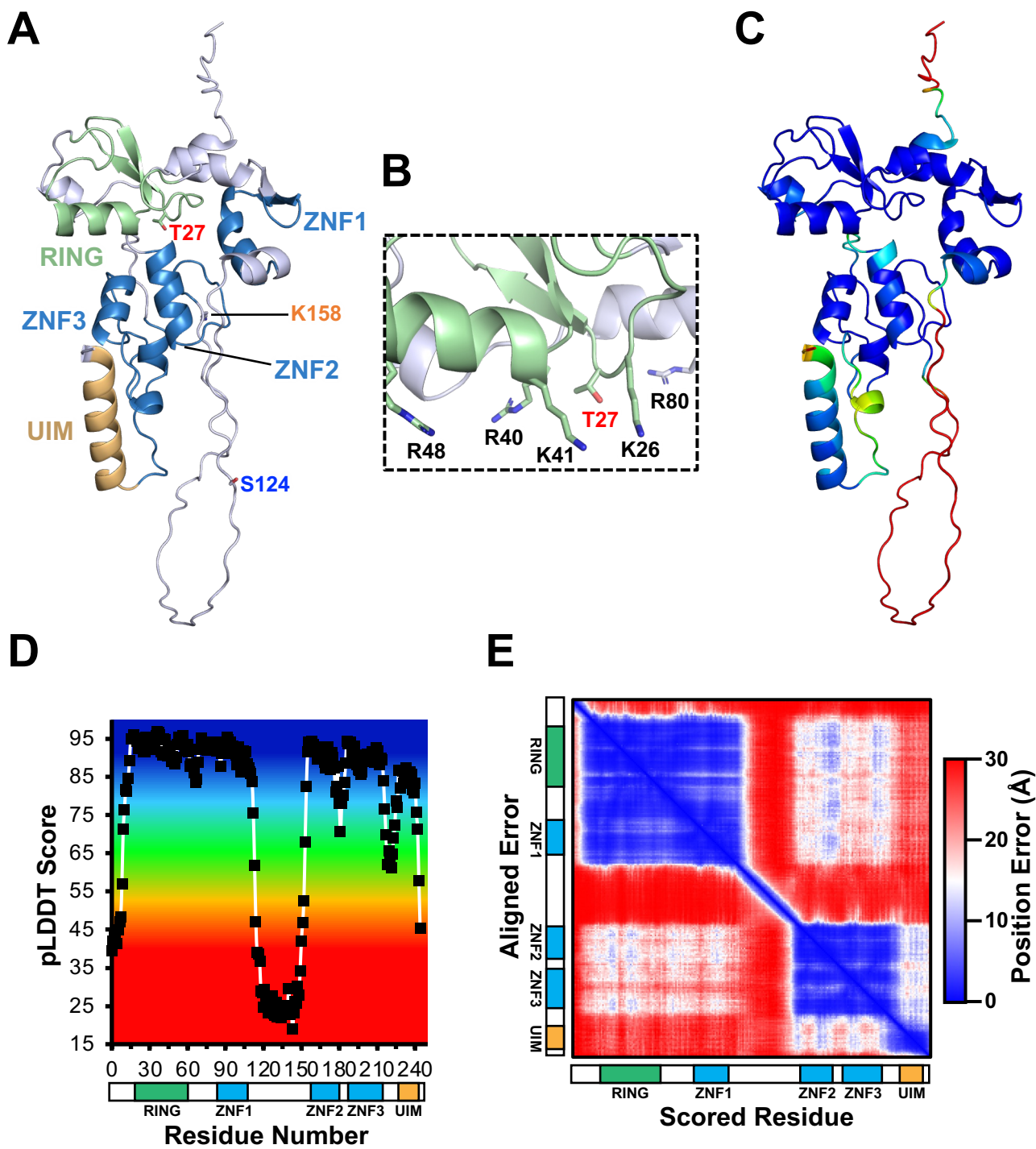

Supplement: Figure S2 — AlphaFold-Predicted Model of RNF138’s Structure.A, predicted model (79, 81, 82) of human RNF138 (UniProt accession Q8WVD3) with individual domains coloured respectively: linkers in white, RING in green, zinc fingers (ZNF) in blue, ubiquitin interacting motif (UIM) in dark yellow. The RING domain folds onto ZNF1, linked together by a hinge-like linker. A longer linker (54 residues) connects the N-terminal RING and ZNF1 to ZNF2 and ZNF3, which appear to pack and form a folded structure and are followed by the UIM. The post-translational modification sites described in this study are highlighted: T27 (red), S124 (dark blue), and K158 (orange). B, closeup view of residue T27 in (A), highlighting positive residues in close proximity and forming a positively charged surface in the RING domain. C and D, Predicted Local Distance Difference Test (pLDDT) (84) plot of the predicted model of RNF138. pLDDT scores range from 0 to 100, where scores above 70 indicate a high level of confidence in the position of the Cɑ atom, and scores above 90 indicate a high level of confidence in the placement of side chain atoms. Scores below 50 are generally considered as indications of low confidence and suggest disorder (84). Dark blue and red correspond to high confidence (>90) and low confidence (<50) pLDDT scores, respectively. The colour gradient on the graph (D) corresponds to the same colour scheme in panel (C). Overall, the model was predicted with high confidence with a median pLDDT of 88.9. The residues in the N-terminal RING domain and ZNF1 had consistently high pLDDT high values. Notably, T27 had a score of 91.6. The linker connecting the N-terminal domains to the C-terminal domains was predicted with low confidence, suggesting flexibility within those residues. S124 is positioned in the flexible linker with a pLDDT score of 26.05, suggesting a dynamic or flexible region (79, 80, 81, 84). The C-terminal zinc fingers and UIM were predicted with high confidence. Interestingly, resid [file mmc2.pdf]

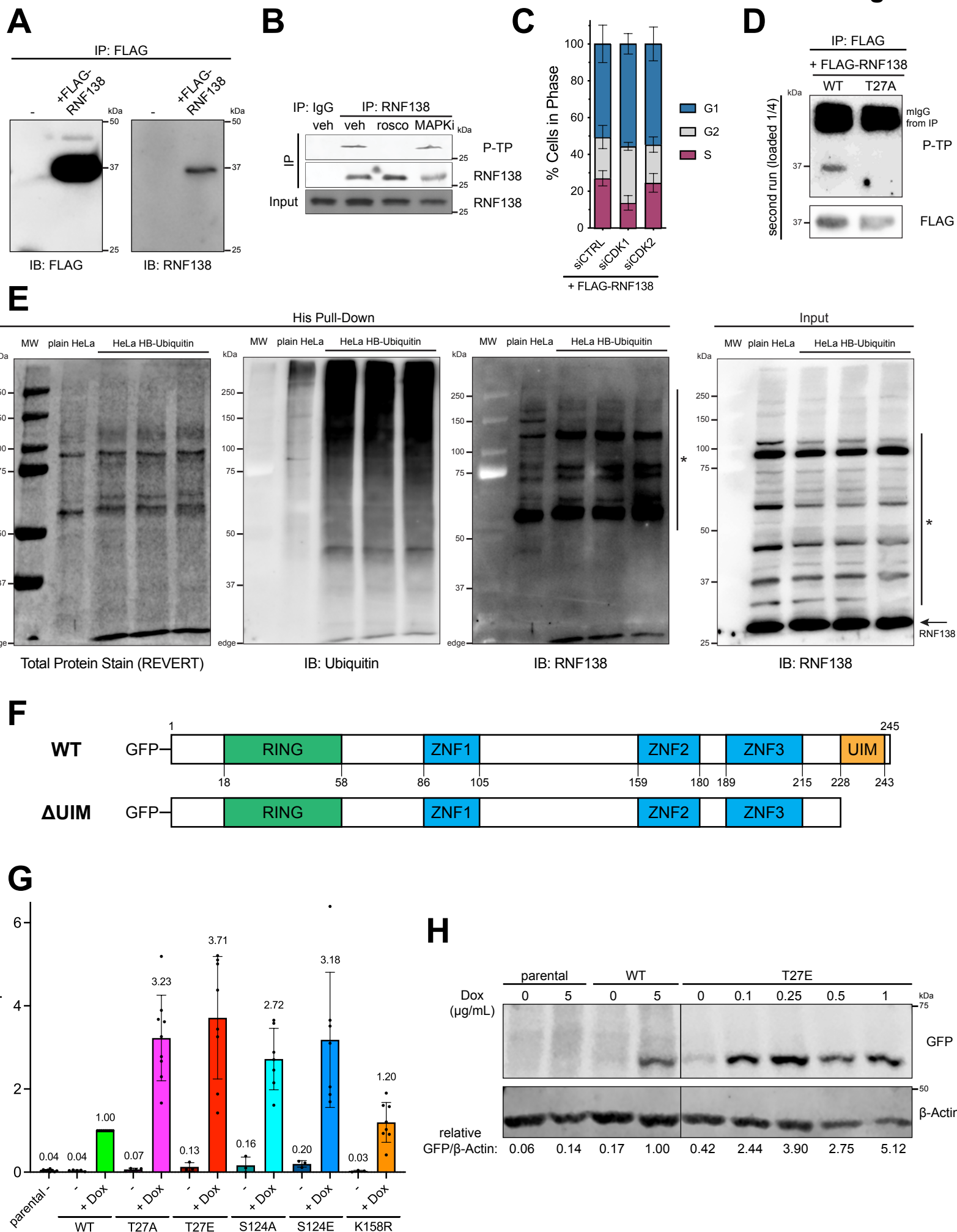

Supplement: Figure S3 — Related toFigure 1, Figure 2and5.A, FLAG immunoprecipitates (IPs) of HEK293 cells expressing FLAG-RNF138 or not were immunoblotted (IB'd) for the FLAG epitope or RNF138 (Abcam antibody). B, G2-synchronized HeLa cells were treated with 0.2% DMSO vehicle control (veh), 50 μM roscovitine (rosco), or the MAP kinase inhibitor SB203580 (MAPKi) at 10 μM for 4 h. Cell extracts were then IP’d for RNF138 or with control immunoglobulin G (IgG) and IB'd for phosphorylated TP sites (P-TP) or RNF138. C, flow cytometric analysis of propidium iodide signal in HeLa cells treated as in Figure 1I. Averages in (C) were calculated from 3 biological replicates pooled together. D, FLAG IP from asynchronous HeLa cells expressing FLAG-RNF138-WT or -T27A and IB'd for P-TP and FLAG. The first run of the IP and the input control is shown in Figure 1L. The second run of the IP is shown here; one-fourth the volume of the IP eluate from the first run is loaded. mIgG: mouse anti-FLAG immunoglobulin G fragments used for IP, detected by the anti-mouse—HRP secondary antibody. E, 90% of a single pellet of plain HeLa cells and 3 cell pellets of HeLa stably expressing 6XHis-biotin-ubiquitin (HeLa HB-ubiquitin) was subjected to nickel affinity purification (“His pull-down”), while the remaining 10% was processed to generate whole cell extract (input). The samples were resolved by SDS-PAGE and transferred to nitrocellulose membrane. The His pull-down fraction was first stained for total protein with REVERT total protein stain (LI-COR Biosciences), then IB’d for RNF138, and finally stripped and IB’d for ubiquitin. For the remaining 10% of cells, whole cell extracts were prepared for the input control (rightmost panel) and IB’d for RNF138. Note the RNF138 antibody detects other species beyond RNF138 (∗). RNF138 itself (predicted molecular weight: 28 kDa) is detected just above 25 kDa (arrow). MW: molecular weight standards. F, schematic diagrams of the structural domains in wildtype (WT) and the ΔUIM mutant [file mmc3.pdf]

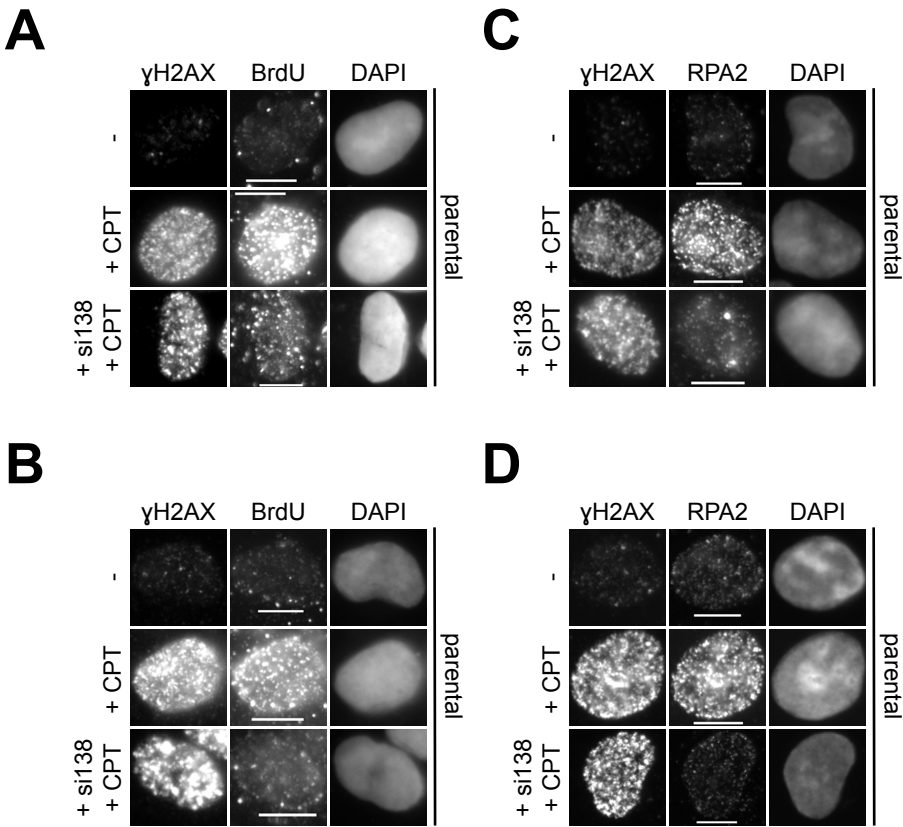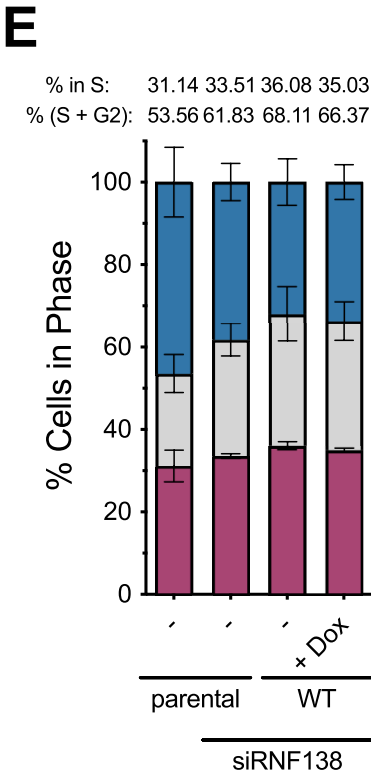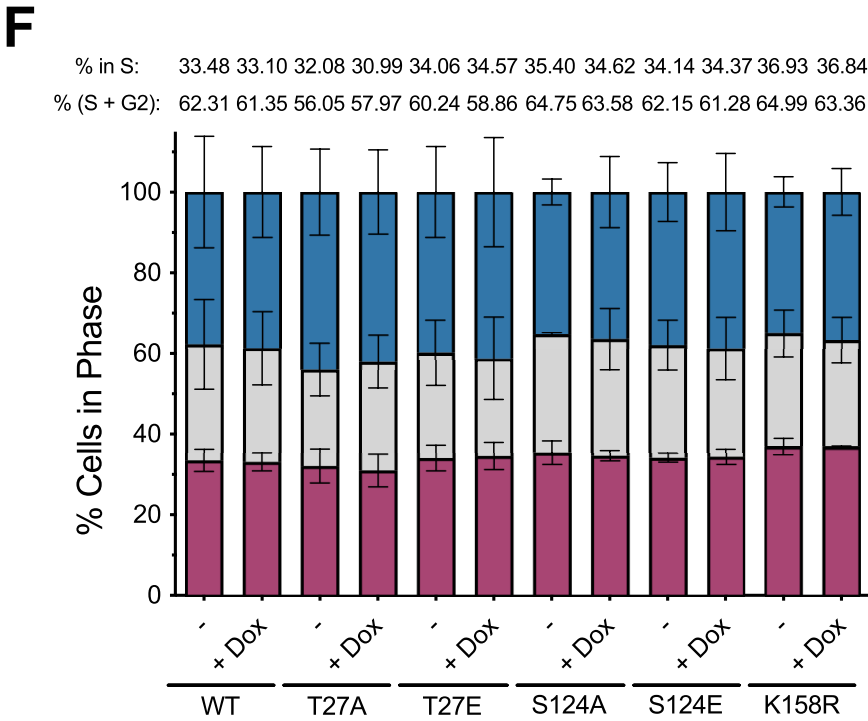

Supplement: Figure S4 — Related toFigures 6and7.A and B, additional representative micrographs from Figure 6. Representative BrdU immunofluorescence (IF) micrographs of parental U2OS-TREx cells transfected with or without siRNA to RNF138 (siRNF138) and treated with camptothecin (CPT) or not for 1 h. ƔH2AX was used to indicate DNA damage, while DAPI stain labeled the nucleus. Micrographs are derived from the same biological replicates presented in the left panels of Figure 6A (A) and Figure 6B (B), respectively. C and D, as per (A and B), but for RPA2 foci instead. Micrographs are derived from the same biological replicates presented in the left panels of Figure 6D (C) and Figure 6E (D), respectively. E, flow cytometric analysis of propidium iodide signal in parental or sfGFP-RNF138-WT-expressing U2OS-TREx cells transfected with siRNA to RNF138 or not, and with or without doxycycline (Dox) induction. F, flow cytometric analysis of propidium iodide signal in sfGFP-RNF138 variant-expressing U2OS-TREx cells induced with Dox or not. Averages were calculated from 2 (F) or at least 2 (E) biological replicates pooled together. Scale bars denote 10 μm. [file mmc4.pdf]
