# Supplementary material for: The role of RNF138 in DNA end resection is regulated by ubiquitylation and CDK phosphorylation
Source: J Biol Chem. 2024 Feb 1;300(3):105709. doi: 10.1016/j.jbc.2024.105709 (PMC10910129; doi:10.1016/j.jbc.2024.105709)
Supplement: Supporting Tables S1–S4 [file mmc5.pdf]

Table S1: Sequencing Primers

Name	Sequence (5' – 3')	Construct	Direction
RNF138 21 For	GGCCACGTCCTACACCGA	any containing RNF138	forward
RNF138 210 For	ACGGGCCTTAGACCTTGAAA	any containing RNF138	forward
RNF138 169 Rev	ATAGGGGACAATGTGCTCCG	any containing RNF138	reverse
FLAG-RNF138 845 Rev	TGAAATTTGTGATGCTATTGCTTT	FLAG-RNF138	reverse
pEGFP-C1 For	CATGGTCCTGCTGGAGTTCGTG	GFP-RNF138	forward
pEGFP-C1 Rev	CAGGTTCAGGGGGAGGTGTGG	GFP-RNF138	reverse

Table S2: Primers for Q5 Site-Directed Mutagenesis (New England Biolabs)

Mutation	Templates	Sequences of Primer Pairs (5' – 3')	Annealing Temp.
**RNF138-T27A**	FLAG-RNF138-WT, GFP-RNF138-WT, mCherry-RNF138-WT	Forward: GGTGCTCAAAgcgCCCGTGCGGA<br>Reverse: TCCTGACAGACGGGGCAGTAG	72°C
**RNF138-T27E**	GFP-RNF138-WT, sfGFP-RNF138-WT, mCherry-RNF138-WT	Forward: GGTGCTCAAAgagCCCGTGCGGAC<br>Reverse: TCCTGACAGACGGGGCAG	67°C
**RNF138-K158R**	GFP-RNF138-WT, mCherry-RNF138 -WT/-T27A/-S124A/ -T27A-S124A	Forward: CCTACTTTTaggTGTCCCCTG<br>Reverse: ATGACCAGAAGAACTTGTATTC	60°C
**RNF138-S124A**	FLAG-RNF138-WT, GFP-RNF138-WT, mCherry-RNF138 -WT/-T27A	Forward: CTTTCAGATCgctCAAGATTCAG<br>Reverse: TTTGGAATGATAGAAGAAACAC	58°C
**RNF138-S124E**	sfGFP-RNF138-WT, mCherry-RNF138-WT	Forward: CTTTCAGATCgagCAAGATTCAGTAGGG<br>Reverse: TTTGGAATGATAGAAGAAACAC	57°C

Table S3: siRNAs

Name	Target	Sense Sequence (5' – 3')
**siCTRL**	Luciferase (negative control)	CGUACGCGGAAUACUUCGA
**siRNF138**	RNF138 coding region (1)	CCAAACUGCUGUUGAAGAA
**siRNF138 UTR** (used only in Fig. S1A)	RNF138 3' UTR (sequences were selected by Dharmacon Inc.)	The following siRNAs pooled together: GGAGGGAAUUGUAUUGAUA AAAGAGUGGUGUUUACUAU GGGAAUAGGGAUAGACUUU AGCCAUACAUCUUAAUGAA
**siCDK1**	CDK1	CCUAGUACUGCAAUUCGGGAAAUUU
**siCDK2**	CDK2	CCUAUUCCCUGGAGAUUCUGAGAUU

All siRNAs were synthesized by Sigma-Aldrich and included [dTdT] overhangs.

REFERENCES

1. Schmidt, C. K., Galanty, Y., Sczaniecka-Clift, M., Coates, J., Jhujh, S., Demir, M., Cornwell, M., Beli, P., and Jackson, S. P. (2015) Systematic E2 screening reveals a UBE2D-RNF138-CtIP axis promoting DNA repair. *Nat Cell Biol*. **17**, 1458–1470

Table S4: Antibodies

Antibody	Source	Catalogue Number	Identifier	Dilution (Application)
rabbit anti-β-actin	Sigma	A5060	RRID:AB_476738	1:10000 – 1:20000 for 1 hour (IB)
mouse anti-BrdU	Cytiva Life Sciences	RPN202	RRID:AB_2314032	1:1000 overnight (IF)
mouse anti-CDK1	Santa Cruz Biotechnology	sc-54	RRID:AB_627224	1:1000 overnight (IB)
rabbit anti-CDK2	Santa Cruz Biotechnology	sc-163	RRID:AB_631215	1:1000 overnight (IB)
rabbit anti-phospho-Chk1 (Ser345)	Cell Signaling Technology	2348	RRID:AB_331212	1:5000 for 1 hour (IB)
rabbit anti-phospho-Chk2 (Thr68)	Cell Signaling Technology	2197	RRID:AB_2080501	1:1000 overnight (IB)
rabbit anti-Cyclin A	Santa Cruz Biotechnology	sc-751	RRID:AB_631329	1:1000 overnight (IB)
mouse anti-FLAG tag	Millipore Sigma	F1804	RRID:AB_262044	1:4000 – 1:8000 for 1 hour (IB)
rabbit anti-FLAG tag	Cell Signaling Technology	14793	RRID:AB_2572291	1:4000 – 1:8000 for 1 hour (IB)
rabbit anti-Geminin	Cell Signaling Technology	5165	RRID:AB_10623289	1:1000 overnight (IB)
mouse anti-GFP	Santa Cruz Biotechnology	sc-9996	RRID:AB_627695	1:2000 – 1:6000 for 1 hour (IB)
rabbit anti-GFP	Proteintech	50430-2-AP	RRID:AB_11042881	1:5000 for 1 hour (IB)
rabbit anti-phospho-H2AX (Ser139)	Cell Signaling Technology	2577	RRID:AB_2118010	1:3000 for 1 hour (IF)
rabbit anti-HA tag	Abcam	ab9110	RRID:AB_307019	1:4000 – 1:8000 overnight (IB)
rabbit anti-RNF138	St John's Laboratory	STJ112342	RRID:AB_2938982	1:1000 – 1:4000 overnight (IB)
rabbit anti-RNF138 (used only in Fig. S3A)	Abcam	ab92730	RRID:AB_2238719	1:1000 overnight (IB)
mouse anti-RPA2	Abcam	ab2175	RRID:AB_302873	1:8000 for 1 hour (IF)<br>1:2000 overnight (IB)

rabbit anti-phospho-RPA2 (Ser4/Ser8)	Bethyl Laboratories	A300-245A	RRID:AB_210547	1:4000 – 1:12000 overnight (IB)
rabbit anti-phospho-Ser/Thr (P-S/TQ; ATM/ATR substrate)	Cell Signaling Technology	2851	RRID:AB_330318	1:1000 overnight (IB)
mouse anti-phospho-Thr-Pro (P-TP)	Cell Signaling Technology	9391	RRID:AB_331801	1:500 – 1:1000 overnight (IB)
mouse anti-α-Tubulin	Genscript	A01410	RRID:AB_1968943	1:20000 – 1:25000 for 1 hour (IB)
mouse anti-Ubiquitin	Santa Cruz Biotechnology	sc-8017	RRID:AB_2762364	1:1000 overnight (IB)
donkey anti-mouse IgG—IRDye 680RD	LI-COR Biotechnology	926-68072	RRID:AB_10953628	1:20000 for 1 hour (IB)
donkey anti-rabbit IgG—IRDye 680RD	LI-COR Biotechnology	926-68073	RRID:AB_10954442	1:20000 for 1 hour (IB)
donkey anti-mouse IgG—IRDye 800CW	LI-COR Biotechnology	926-32212	RRID:AB_621847	1:20000 for 1 hour (IB)
donkey anti-rabbit IgG—IRDye 800CW	LI-COR Biotechnology	926-32213	RRID:AB_621848	1:20000 for 1 hour (IB)
goat anti-mouse IgG—HRP	LI-COR Biotechnology	926-80010	RRID:AB_2721263	1:5000 for 1 hour (IB)
goat anti-rabbit IgG—HRP	LI-COR Biotechnology	926-80011	RRID:AB_2721264	1:5000 for 1 hour (IB)
goat anti-mouse IgG—Cy3	Jackson ImmunoResearch Laboratories	115-165-146	RRID:AB_2491007	1:500 for 1 hour (IF)
goat anti-rabbit IgG—Alexa Fluor 647	Invitrogen	A-21244	RRID:AB_2535812	1:250 for 1 hour (IF)

- IF = immunofluorescence staining; IB = immunoblot
- 1 hour = 1 hour incubation at room temperature is preferable
- Overnight = overnight incubation at 4°C is preferable
